# Hybrid Gene Origination Creates Human-Virus Chimeric Proteins during Infection

**DOI:** 10.1016/j.cell.2020.05.035

**Published:** 2020-06-25

**Authors:** Jessica Sook Yuin Ho, Matthew Angel, Yixuan Ma, Elizabeth Sloan, Guojun Wang, Carles Martinez-Romero, Marta Alenquer, Vladimir Roudko, Liliane Chung, Simin Zheng, Max Chang, Yesai Fstkchyan, Sara Clohisey, Adam M. Dinan, James Gibbs, Robert Gifford, Rong Shen, Quan Gu, Nerea Irigoyen, Laura Campisi, Cheng Huang, Nan Zhao, Joshua D. Jones, Ingeborg van Knippenberg, Zeyu Zhu, Natasha Moshkina, Léa Meyer, Justine Noel, Zuleyma Peralta, Veronica Rezelj, Robyn Kaake, Brad Rosenberg, Bo Wang, Jiajie Wei, Slobodan Paessler, Helen M. Wise, Jeffrey Johnson, Alessandro Vannini, Maria João Amorim, J. Kenneth Baillie, Emily R. Miraldi, Christopher Benner, Ian Brierley, Paul Digard, Marta Łuksza, Andrew E. Firth, Nevan Krogan, Benjamin D. Greenbaum, Megan K. MacLeod, Harm van Bakel, Adolfo Garcìa-Sastre, Jonathan W. Yewdell, Edward Hutchinson, Ivan Marazzi

**Affiliations:** 1 Department of Microbiology, Icahn School of Medicine at Mount Sinai, New York, NY 10029, USA; 2 Laboratory of Viral Diseases, National Institute of Allergy and Infectious Diseases, NIH, Bethesda, MD 20892, USA; 3 Department of Cellular and Molecular Pharmacology, University of California, San Francisco, San Francisco, CA 94158, USA; 4 Department of Medicine, School of Medicine, University of California San Diego, La Jolla, CA 92037, USA; 5 Department of Genetics and Genomic Sciences, Icahn School of Medicine at Mount Sinai, New York, NY 10029, USA; 6 Tisch Cancer Institute, Icahn School of Medicine at Mount Sinai, New York, NY 10029, USA; 7 Department of Medicine, Hematology and Medical Oncology, Icahn School of Medicine at Mount Sinai, New York, NY 10029, USA; 8 Department of Oncological Sciences, Icahn School of Medicine at Mount Sinai, New York, NY 10029, USA; 9 Department of Pathology, Icahn School of Medicine at Mount Sinai, New York, NY 10029, USA; 10 Divisions of Immunobiology and Biomedical Informatics, Cincinnati Children’s Hospital, Cincinnati, OH 45229, USA; 11 Department of Pediatrics, University of Cincinnati College of Medicine, Cincinnati, OH 45257, USA; 12 Global Health and Emerging Pathogens Institute, Icahn School of Medicine at Mount Sinai, New York, NY 10029, USA; 13 Division of Infectious Diseases, Department of Medicine, Icahn School of Medicine at Mount Sinai, New York, NY 10029, USA; 14 MRC-University of Glasgow Centre for Virus Research, Glasgow G61 1QH, UK; 15 Instituto Gulbenkian de Ciência, 2780-156 Oeiras, Portugal; 16 The Roslin Institute, University of Edinburgh, Edinburgh EH25 9PS, UK; 17 Division of Virology, Department of Pathology, University of Cambridge, Cambridge CB2 0SP, UK; 18 Centre for Immunobiology, Institute of Infection, Immunity and Inflammation, University of Glasgow, Glasgow G12 8QQ, UK; 19 Department of Pathology, the University of Texas Medical Branch, Galveston, TX 77555, USA; 20 Division of Structural Biology, The Institute of Cancer Research, London SW7 3RP, UK; 21 Fondazione Human Technopole, Structural Biology Research Centre, 20157 Milan, Italy

**Keywords:** gene origination, influenza, uORFs, viral evolution, chimeric proteins, segmented negative-strand RNA viruses, cap-snatching, upstream AUG, viral RNA, RNA hybrid

## Abstract

RNA viruses are a major human health threat. The life cycles of many highly pathogenic RNA viruses like influenza A virus (IAV) and Lassa virus depends on host mRNA, because viral polymerases cleave 5′-m7G-capped host transcripts to prime viral mRNA synthesis (“cap-snatching”). We hypothesized that start codons within cap-snatched host transcripts could generate chimeric human-viral mRNAs with coding potential. We report the existence of this mechanism of gene origination, which we named “start-snatching.” Depending on the reading frame, start-snatching allows the translation of host and viral “untranslated regions” (UTRs) to create N-terminally extended viral proteins or entirely novel polypeptides by genetic overprinting. We show that both types of chimeric proteins are made in IAV-infected cells, generate T cell responses, and contribute to virulence. Our results indicate that during infection with IAV, and likely a multitude of other human, animal and plant viruses, a host-dependent mechanism allows the genesis of hybrid genes.

## Highlights

• A mechanism of hybrid gene birth is employed by many families of RNA viruses
• Human RNA and viral RNA encode new genes together
• Hybrid genes either make extensions of viral proteins or novel proteins (UFOs)
• Human-virus genes and proteins play roles in pathogenesis and are conserved

## Graphical Abstract

**Figure:**
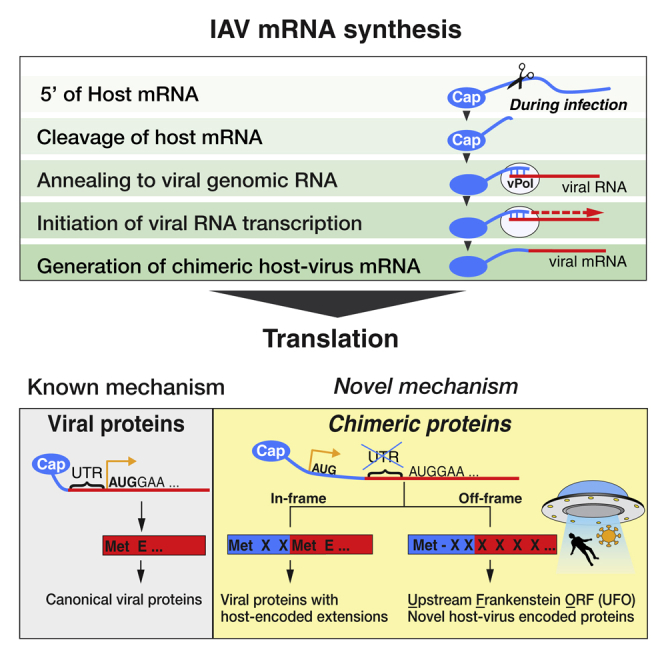

## Teaser

The process by which RNA viruses, such as influenza virus, cleave capped host transcripts to drive viral mRNA production leads to the translation of host and viral RNA to make hybrid proteins that then generate T cell responses and contribute to virulence.

## Introduction

In eukaryotes, ribosomes typically recognize mRNAs with a terminal 5′ cap structure followed by an untranslated region (UTR), which can be tens to hundreds of nucleotides in length (Decroly et al., 2011, Kochetov et al., 2008, Leppek et al., 2018). However, a growing body of work has shown that translation can initiate in the 5′ UTRs of a large proportion of eukaryotic mRNAs, sometimes extremely close to the 5′ cap, resulting in upstream open reading frames (uORFs) (Andreev et al., 2015, Calvo et al., 2009, Dikstein, 2012, Elfakess and Dikstein, 2008, Haimov et al., 2017, Johnstone et al., 2016, Kochetov et al., 2008, Young and Wek, 2016).

A large subphylum of RNA viruses, the segmented negative strand RNA viruses (sNSVs), makes direct use of the 5′ termini of host mRNAs when transcribing their own genes. The sNSVs include the families *Arenaviridae*, *Peribunyaviridae*, and *Orthomyxoviridae.* Highly contagious human and animal viruses like influenza A virus (IAV) and Lassa virus (LASV) belong to these families and are responsible for significant levels of morbidity and mortality worldwide. In sNSVs, viral mRNA synthesis is primed using short 5′ methyl-7-guanosine (m^7^G) capped RNA sequences, which the viral polymerase cleaves from host RNA polymerase II (RNAPII) transcripts in a process known as “cap-snatching” (Dias et al., 2009, Plotch et al., 1981, Reich et al., 2014, Rialdi et al., 2017). Cap-snatching creates viral transcripts that are genetic hybrids of host and viral sequences, with the host-derived 5′ sequences being highly diverse (Gu et al., 2015, Koppstein et al., 2015, Rialdi et al., 2017, Sikora et al., 2017). Once made, viral mRNAs are translated by the host machinery.

In this manuscript, we hypothesized that by appropriating 5′ terminal mRNA sequences from their hosts, sNSVs could obtain functional upstream start codons (uAUGs), a mechanism we termed “start-snatching.” Translation from host-derived upstream start codons in chimeric host-viral transcripts would access upstream viral ORFs (uvORFs). Depending on the frame of the uAUG relative to that of the canonical viral protein, two novel chimeric types of protein in infected cells could be generated: canonical viral proteins with host and viral UTR-derived N-terminal extensions, and previously uncharacterized proteins read from ORFs that are out-of-frame with, and overprinted on, canonical viral ORFs. Below, we report on how we tested this hypothesis using genomics, cell biology, virology, and phylogenetic analyses.

## Results

### IAV Cap-Snatches Sequences Containing uAUGs

**Figure 1:**
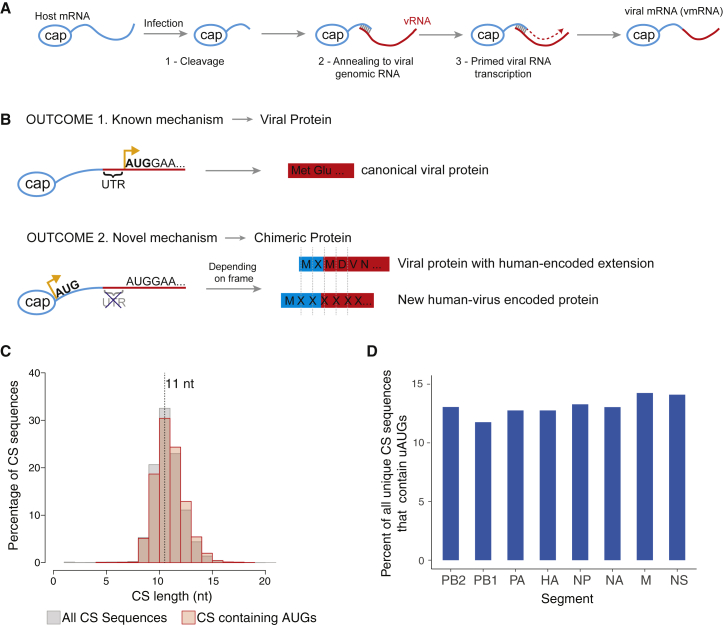
Upstream AUGs Are Present in Host-Derived Sequences of Viral mRNAs (A) Schematic of cap-snatching during the transcription of a segmented negative sense RNA virus (sNSV) such as influenza A virus (IAV). (B) Schematic showing how the presence of upstream AUGs (uAUGs) in host-derived cap-snatched RNA sequences may drive the formation of novel host-viral chimeric proteins. (C) Histograms showing the length distributions of all cap-snatched (CS) sequences (gray bars) or only CS sequences containing uAUGs (red bars) in A549 cells infected with IAV (strain PR8) for 4 h, as determined by DEFEND-seq. (D) Bar plots showing the percentages of uAUG containing CS sequences in each IAV genome segment.

IAV gene transcription is initiated by cap-snatching from a host mRNA (Figure 1A). This process generates an IAV mRNA with a 5′ end portion derived from the host. This mechanism is used to express viral genes that encode canonical viral proteins (Figure 1B, OUTCOME 1). We hypothesized that AUGs within host sequences could generate upstream host-virus chimeric ORFs with coding potential. Depending on the reading frame, a host-derived uAUG might initiate the synthesis of two novel chimeric genes encoding for an N-terminally extended viral protein (Figure 1B, OUTCOME 2, upper panel) or alternatively, an entirely novel protein overprinted on the canonical viral ORF (Figure 1B, OUTCOME 2, lower panel). These outcomes are contingent on two assumptions: (1) uAUGs are present in cap-snatched host sequences and can enable translation initiation, and (2) the 5′ mRNA transcribed from the viral UTR should lack stop codons. Furthermore, the absence of stop codons interrupting UTRs or the downstream ORFs should be evolutionarily conserved.

**Figure S1:**
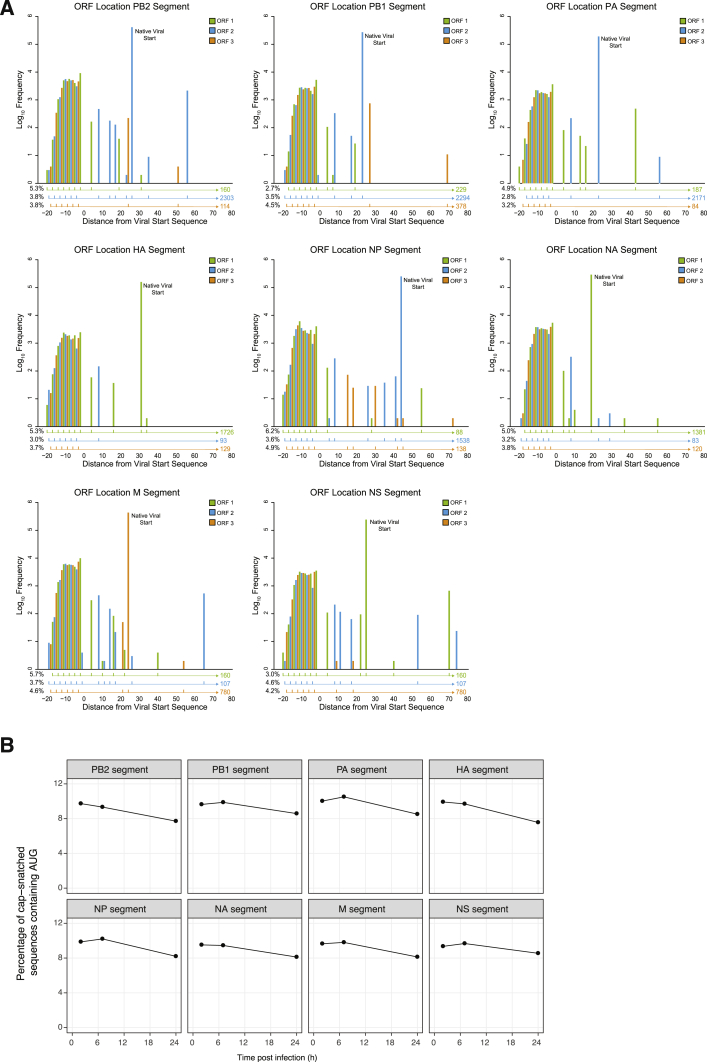
uAUGs Are Present in Viral mRNAs, Related to Figure 1 (A) Incorporation of host transcript sequences increases the diversity of putative alternative start codons. For each viral genome segment, the frequency and position of alternative start codons is shown relative to native start of the viral genes. For each reading frame, the frequency and location of the first in-frame stop codon are indicated. (B) Percentages of cap-snatched sequences that contain AUG codons, as identified by CAGE. Data are shown relative to all the viral reads from the specified genome segments.

To address the first point, we determined the abundance of uAUGs in cap-snatched host sequences archived in a Decap and 5′ end sequencing (DEFEND-seq) dataset (Rialdi et al., 2017) that we had previously generated from A549 cells infected with the IAV A/Puerto Rico/8/34(H1N1) (PR8) (Figure 1C). AUG-containing, host-derived capped sequences (Figure 1C, red bars) ranged from 7–20 nt, with a median length of 11 nt, similar to the distribution obtained for all cap-snatched sequences (Figure 1C, gray bars). Host-derived oligonucleotides with AUG codons were present at similar ratios in all eight genome segments of the virus and were present in all three reading frames, constituting ∼12% of all cap-snatched sequences (Figures 1D and S1A). Similar results were also obtained when we performed cap analysis of gene expression (CAGE) on primary human monocyte-derived macrophages infected with a different strain of IAV (A/Udorn/72(H3N2); Udorn) (Figure S1B; Table S1). These results indicate that, upon infection, neither the virus nor the host cells appear to prevent the formation of chimeric RNAs with hybrid coding potential.

### IAV 5′ UTRs Are Translatable

**Figure S2:**
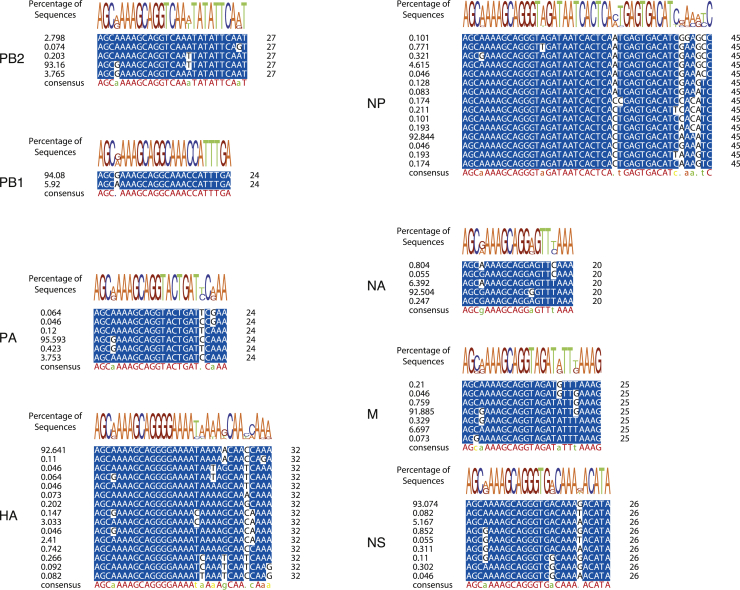
Viral 5′ UTRs Are Conserved, Related to Figure 2 Multiple sequence alignments of unique H1N1 IAV 5′UTRs per genome segment (n = 10904). The overall distribution of each unique nucleotide sequence is indicated on the left, and the consensus sequence of each UTR is indicated below each alignment. The top panels show the positional weight matrix of each nucleotide across the UTRs.

**Figure 2:**
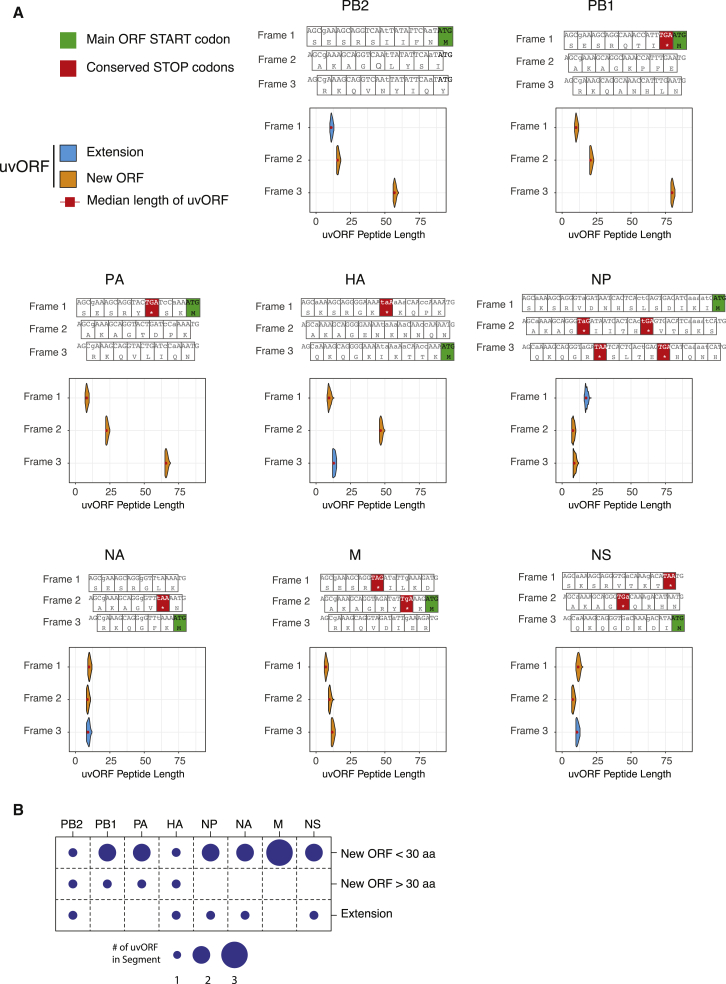
IAV 5′ UTRs Are Conserved and Translatable (A) Sequence analysis of all unique 5′ UTR sequences from each segment of 10,904 H1N1 subtype IAV genomes (coding sense), showing (upper panels) the translation of the 5′ UTR in all three reading frames; and (lower panels) the predicted amino acid length (aa) distributions of N-terminal extensions to the major gene product and of overprinted new ORFs. This is calculated from the distribution of uAUG positions in DEFEND-seq data and (for overprinted new ORFs) from the position of stop codons in the IAV PR8. (B) The numbers of translatable products that could be accessed from uAUGs in each genome segment of IAV.

We next performed a bioinformatic analysis to determine if stop codons were absent from IAV sequences within the 5′ UTRs and, if so, whether this was evolutionarily conserved across IAV strains. First, we analyzed the nucleotide sequence variability of the 5′ UTRs of all eight segments, using all IAV H1N1 strains available from the NCBI Influenza Virus database (Zhang et al., 2017). 5′ UTRs of each individual segment are highly conserved within each individual segment, as shown by the positional weight matrices (Figure S2, top panels) and sequence alignment (Figure S2, lower panels). We then translated the 5′ UTR of each genome segment *in silico* in all possible frames (Figure 2A, upper panels) This revealed that the 5′ UTR of every IAV genome segment can maintain a reading frame in at least one frame (Figure 2A, upper panels, stop codons indicated by red boxes).

We found that the 5′ UTRs of five out of the eight genome segments (PB2, HA, NP, NA, and NS) lacked upstream stop codons in-frame with the major ORF (Figure 2A, upper panels, major ORF start codons indicated by green boxes). These segments thus have the potential to code for N-terminally extended viral proteins. Stop codons were also absent from the 5′ UTRs of six of the eight genome segments when these were read out of frame with the major ORF (Figure 2A; segments PB2, PB1, PA, NA, M, and HA). This suggested the intriguing possibility that, in the presence of a host-donated start codon, these genome segments could make novel genes encoding hybrid polypeptides.

To probe the length of uvORFs, we translated viral sequences that had cap-snatched uAUGs in our dataset *in silico.* The result of these analyses (Figure 2A, lower panels) indicated the general propensity to create chimeric ORFs, with half of the viral genome segments predicted to make sizable products (>30 aa) (Figure 2B). These ORFs overlap with canonical viral genes but are read in different frames (overprinted). They range from over 40 residues (HA) to nearly 80 residues (PB1). Where N-terminal extensions of the major ORF were possible, these ranged from ∼8–21 aa in length (Figure 2B).

Thus, uvORFs are present in all genome segments and, if licensed by host-derived uAUG-containing RNAs, could generate polypeptides of varying length (Figure 2B).

### Host-Virus mRNA Chimeras Associate with Elongating Ribosomes

**Figure 3:**
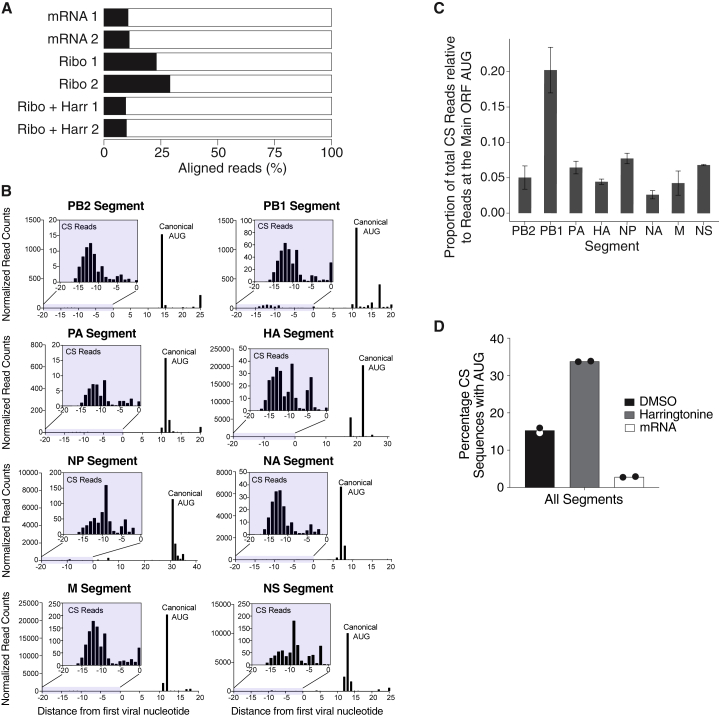
IAV mRNAs Can Be Translated from Host-Derived AUGs (A) Proportion of reads that align to viral and human transcripts for the indicated experimental conditions. (B) 5′ end mapping of ribosome protected fragments (RPFs) in harringtonine-treated A549 cells infected with the IAV PR8 at 8 h post-infection, showing for each segment of the IAV genome the distribution of reads in the cap-snatched regions (shown in insets) and virally encoded mRNA up to 10 nt after the canonical start codon. The x axis is shown relative to the first virally encoded nucleotide. (C) For each IAV genome segment, the number of ribosome-protected fragments (RPFs) upstream of the canonical AUG as a proportion of those mapping to the canonical AUG is shown. Data are shown as the mean ± SD. (D) Barplots showing the percentages of RPFs that contain an AUG when cells were treated with DMSO (black bars) or harringtonine (gray bars) immediately prior to harvest, or from total mRNA-seq (white bars). Results from two sequencing replicates are shown as points, with bars showing the mean.

**Figure S3:**
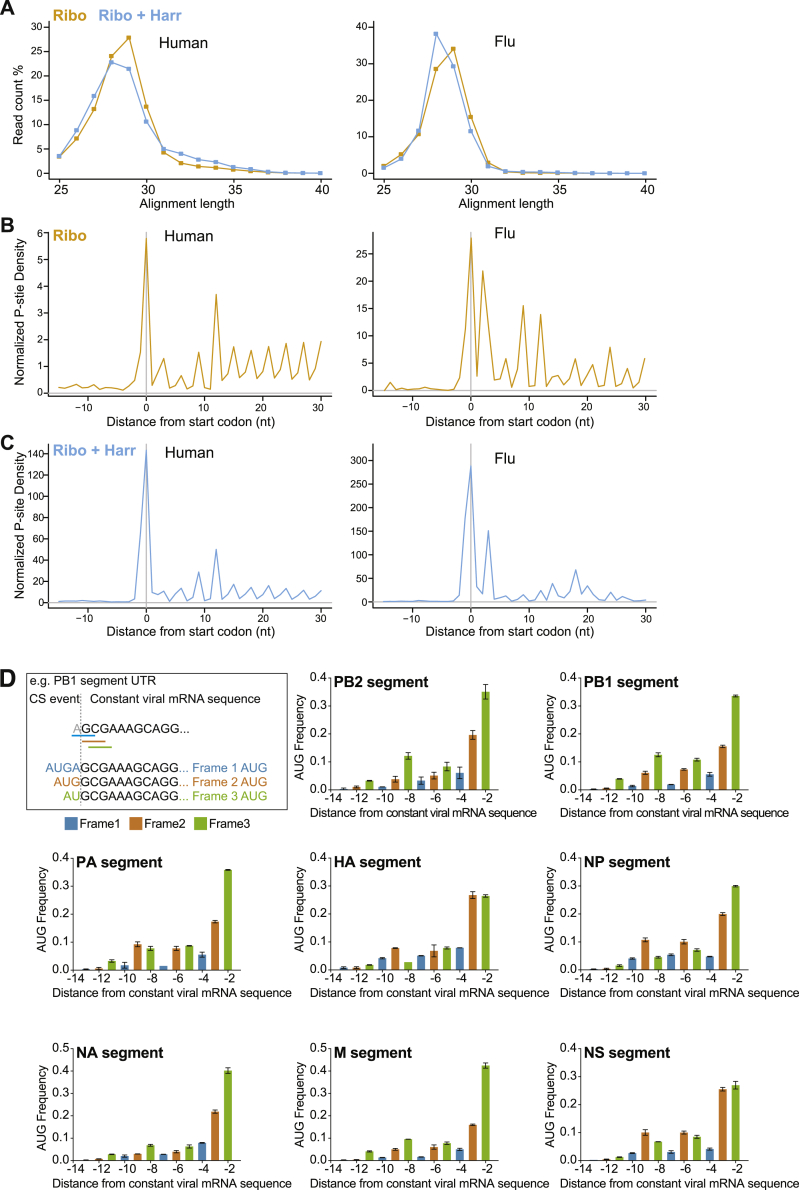
IAV mRNAs Can Be Translated from Host-Derived AUGs, Related to Figure 3 (A) Length distribution of ribosome profiling reads that aligned to human (left panel) and viral (right panel) transcripts in DMSO (Ribo) or harringtonine (Ribo + Harr) treated samples. (B) Metagene alignment of average P site density around annotated start codons in human (left panel) or viral (right panel) transcripts in DMSO treated samples. (C) Metagene alignment of average P site density around annotated start codons in human (left panel) or viral (right panel) transcripts in harringtonine treated samples. (D) Frequency of AUG codons by position relative to the viral transcription initiation site. Bars show the mean frequency and are color coded according to frame. Error bars indicate the standard deviation.

If cap-snatched host uAUGs did initiate translation of viral 5′ UTRs, the 5′ termini of viral mRNAs would be bound by initiating ribosomes. We therefore performed ribosomal profiling of IAV infected cells, in the presence of harringtonine, which blocks elongation of *de novo* assembled 80S initiation complexes but not of those already engaged in elongation. Ribosome-protected fragments (RPFs) were mapped to both the human and viral genomes (Figures 3A and S3A–S3C). Mapping of RPF sequences revealed an accumulation of ribosomes at the canonical initiation site in mRNAs transcribed from all eight genome segments (Figure 3B; main ORF AUG), consistent with previous reports (Machkovech et al., 2019). As well as observing ribosomes accumulating at the canonical initiation sites, we also observed RPFs mapping to the host-derived sequence upstream of the 5′ UTR, suggesting that translation initiated in this region (Figure 3B, insets). The total number of RPF reads mapping to host-derived sequences for each segment was 5%–20% of the reads mapping to the canonical start codon (Figure 3C), broadly consistent with the proportion of cap-snatched sequences containing uAUGs (Figures 1D and S1B).

Precisely mapping initiation sites very close to the cap is challenging, because many of the heterogeneous 5′ mRNA ends would be too short to extrude from the ribosome, making P-site phasing problematic by standard Ribo-seq analysis. To address this, we used the location of AUGs within the RPF to identify the reading frame being translated. This suggested that initiation occurred in all three reading frames (Figure S3D). uAUG codons were more frequently close to the start of the viral UTR sequences, peaking at the −2 position of mRNAs from all genome segments (numbered from the first position in the coding sense of the viral genome segment), and less frequent toward the 5′ end of the host-derived sequence (Figure S3D). As well as inferring upstream ribosome initiation by mapping RPFs to protected uAUGs, we could test for it directly by comparing ribosomal profiles with and without harringtonine arrest. Harringtonine increased the proportion of RPFs from cap-snatched sequences that contained an AUG, indicating translation was initiating on uAUGs in these host-derived sequences (Figure 3D). Taken together, our data show that translation initiates from cap-snatched host-derived uAUGs in viral mRNA chimeras, albeit at lower frequencies than at canonical start codons.

### Host-Virus Protein Chimeras Are Expressed during Infection, Recognized by T Cells, and Affect Virulence

**Figure 4:**
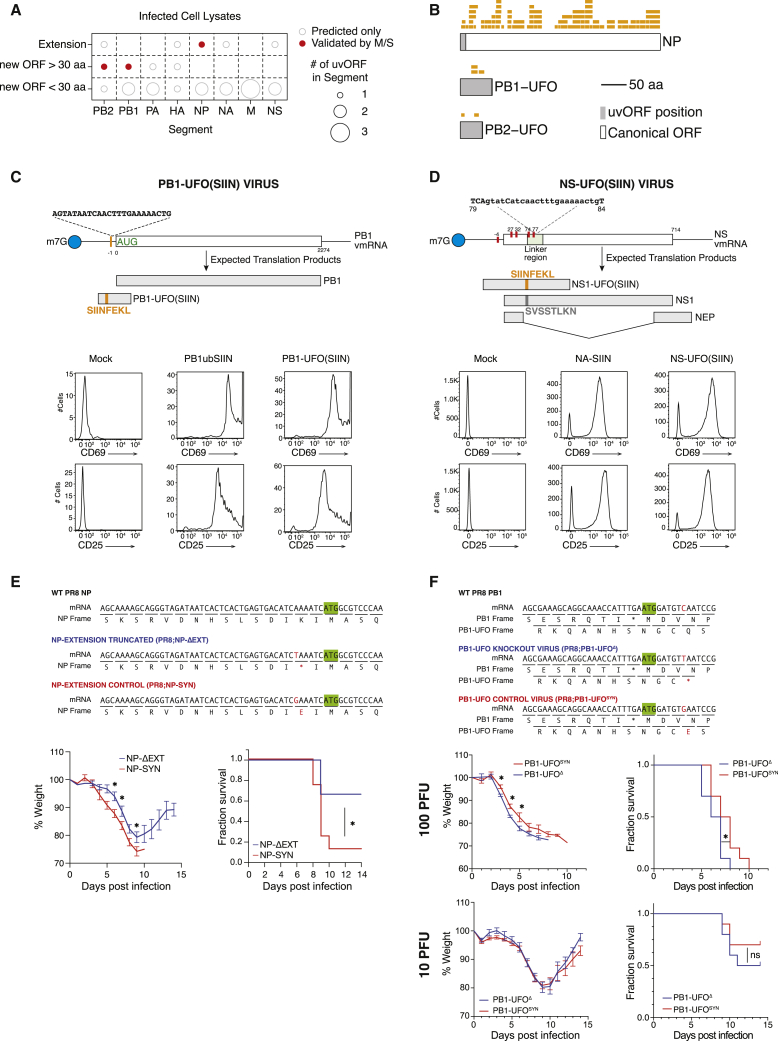
uvORFs Are Expressed during Infection and Can Contribute to Virulence (A) The number of upstream viral open reading frames (uvORFs) that could be translated for each segment of the IAV genome (empty circles), highlighting those detected in infected cell lysates by mass spectrometry (filled red circles). (B) Tryptic peptides that map to translated uvORFs, detected by mass spectrometry across multiple experiments (summarizing data in Figures S4A and S4C). (C) Schematic showing the generation of the PB1-UFO(SIIN) virus. DC2.4 cells were infected with the indicated viruses and co-cultured with OT-I CD8^+^ T cells. OT-1 activation, assessed by CD69 and CD25 expression, was assayed by flow cytometry at 24 h post co-culture. vmRNA, viral mRNA. (D) Schematic showing the generation of the NS-SIIN virus. Red bars indicate stop codons mutated to permit uninterrupted NS1-UFO translation. Mouse BMDC cells were incubated with IAV antigen presentations, and co-cultured with OT1-CD8^+^ T cells. OT-I activation, assessed by CD69 and CD25 expression, was assayed by flow cytometry of CD69 and CD25 expression at 24 h post co-culture. (E) Upper panel: schematic showing mutations that truncate NP-ext (NP-ΔEXT) and control mutations (NP-SYN), as engineered into the IAV PR8. Wild-type PR8 is also shown. Lower panel: weight loss and survival curves of 6- to 8-week-old BALB/c mice infected with 15 plaque-forming unit (PFU)/mouse of the indicated viruses. Data are an aggregate of 2 independent experiments of n = 3 mice, using 2 independently plaque purified clones of the NP-ΔEXT or PR8;NP-SYN viruses (total n = 6/condition). ^∗^p < 0.05; data are shown as the mean ± SEM. (F) Upper panel: schematic showing mutations that knocked out PB1-UFO (PB1-UFO^Δ^) and control mutations (PB1-UFO^SYN^), as engineered into the IAV PR8. Wild-type PR8 is also shown. Lower panel: weight loss and survival curves of 6- to 8-week-old BALB/c mice infected with the indicated dose (per mouse) of the indicated viruses. n = 10 mice/condition. ^∗^p < 0.05. Data are shown as the mean ± SEM.

**Figure S4:**
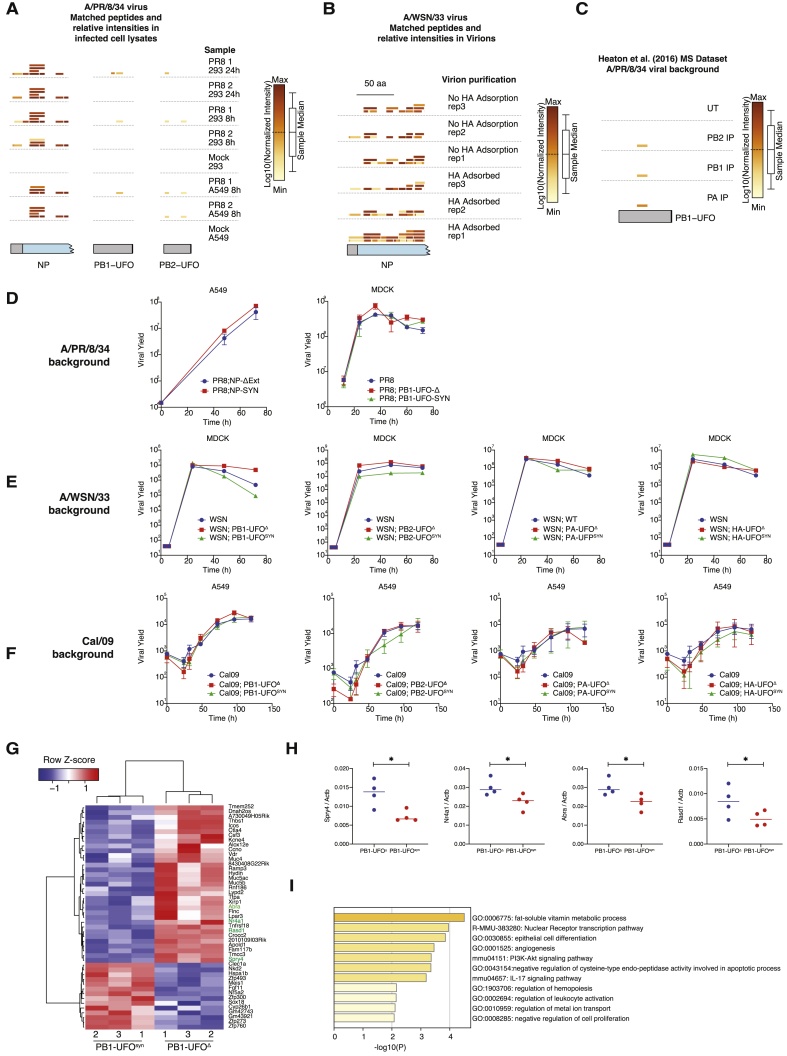
uvORFs Are Expressed during Infection and Can Contribute to Virulence, Related to Figure 4 (A) Plots showing the position of uvORF peptides found in lysates of cells (A549 or 293) infected with A/PR/8/34 virus at 8 or 24h post infection. The specific cell lysates they were found in are indicated on the right. 1: MG132 treated, 2: DMSO treated. Peptide locations are drawn relative to uvORFs (gray regions) and canonical ORFs (blue regions) and are colored by the log_10_ of their intensities, relative to the sample median. (B) Same as in (A), but for uvORF peptides found within purified A/WSN/33 virions. (C) Same as in (A), but for uvORF peptides found from an independent, previously published dataset. (D) *In vitro* growth curves of the indicated mutant (UFO^Δ^) and control (UFO^SYN^) viruses made in the PR8 background, and performed on MDCK cells. Error bars indicate the standard deviation of 3 replicates. (E) *In vitro* growth curves of the indicated mutant (UFO^Δ^) and control (UFO^SYN^) viruses made in the WSN/33 background, and performed on MDCK cells. (F) *In vitro* growth curves of the indicated mutant (UFO^Δ^) and control (UFO^SYN^) viruses made in the Cal/09 background, and performed on A549 cells. Error bars indicate the standard deviation of 3 replicates. (G) Heatmap of differentially expressed genes (Fold Change > 2, p < 0.01) found in the lungs of mice infected with 100PFU of either the PR8;PB1-UFO^Δ^ or PR8;PB1-UFO^SYN^ viruses at day 6 post infection. (H) qPCR validation of four significantly changed genes identified in (G) (highlighted with green text). Each dot represents the lung of one mouse infected with 100PFU of the indicated viruses, collected at day 6 post infection. P values were calculated through a one tailed t test. ^∗^p < 0.05 (I) Gene ontology analysis of genes shown in (G).

To demonstrate that chimeric proteins are expressed during infection, we performed mass spectrometry analyses of cell lysates from infected cells. We also checked whether any chimeric proteins could be integrated into viral progeny by analyzing purified virions (Figures 4A, S4A, and S4B).

There are limitations to this approach, as the likelihood of a tryptic digest generating peptides that can be detected by the mass spectrometer is lower for short proteins. This issue reduces the chance of finding peptides derived from small overprinted uvORFs (<30 aa), or that map to short N-terminal extensions. Nevertheless, we were able to identify at least 2 distinct peptides that were derived from the two long overprinted uvORFs in the PB1 and PB2 segments, which we named PB1-UFO and PB2-UFO, respectively (for “Upstream Frankenstein ORF”). In addition, we detected a UTR-encoded N-terminal extension of NP, which we named NP-extension (NP-ext) (Figures 4A, 4B, S4A, and S4B; Table S2A). Peptides from all three proteins were present in PR8 IAV infected cell lysates (Figures 4B, left panels, and S4A; Table S2A). These novel viral peptides were not detected in uninfected controls (Figure S4A). We were also able to identify peptides derived from the PB1-UFO protein when we re-analyzed three previously published proteomic datasets of IAV infection (Heaton et al., 2016) (Figure S4C; Table S2C). Only NP-ext was detected in virions (Figure S4B; Table S2B), presumably because influenza virions specifically package hundreds of copies of NP, while there is no known mechanism to specifically package other uvORF-encoded proteins (Hutchinson et al., 2014).

Quantification of the PB1-UFO, PB2-UFO, and NP-ext proteins indicated that, although they are less abundant than the major viral proteins, they are expressed at detectable levels within an infected cell. When quantified, tryptic peptides from these proteins were found between the 20^th^ and 40^th^ percentile of normalized peptide intensities, including both host and viral proteins, within our samples (Figures S4A and S4B). Taken together, our data show that N-terminal extensions and overprinted uvORFs are synthesized during IAV infection and are present at a moderate abundance within infected cells.

We next asked whether chimeric host-viral proteins could be recognized by the host’s immune system. To test this, we created modified IAVs containing insertions of a class I-restricted epitope of ovalbumin (Porgador et al., 1997). Based on the uvORFs predicted from our *in silico* analyses, we inserted the epitope (OVAI; OVA 257-264; SL8; SIINFEKL) in frame with the longest uvORF (PB1 frame 3 uvORF; PB1-UFO(SIIN) (Figure 4C) and one of the shortest uvORFs (NS, frame 2 uvORF; NS-UFO(SIIN) (Figure 4D). In the case of PB1 segment, we integrated sequences encoding OVAI directly into the UTR, placing the epitope within the uvORF encoding PB1-UFO (Figure 4C, top panels). For the NS segment, we used synonymous mutations in the canonical viral gene to delete five naturally occurring stop codons in the uvORF; we then inserted OVAI into the extended uvORF, positioning the insertion in a flexible “linker” region of the major viral gene NS1 (Thulasi Raman and Zhou, 2016). This genetic configuration was chosen to ascertain whether uvORFs are translated by default provided that they are not interrupted by stop codons (Figure 4D, top panels).

Mouse DC2.4 cells infected with PB1-UFO(SIIN) activated transgenic OT-I CD8^+^ T cells (that are highly specific for mouse H-2 K^b^ class I molecule complexed with SIINFEKL; Kb-SIIN) (Hogquist et al., 1994) as determined by upregulation of CD25 and CD69 (Figure 4C, lower panels). Recombinant IAV expressing SIIN(PB1-Ub-SIIN) at high levels (Wei et al., 2019) was used as a positive control (Figure 4C, right panels). No upregulation of CD25 and CD69 was observed in mock treated samples. Similar results were obtained with the NS-UFO(SIIN) virus. Here, OT-I CD8^+^ T cells were activated when incubated with bone marrow-derived dendritic cells (BMDCs) infected with the NS-UFO(SIIN) virus (Figure 4D, right panels). This was comparable to the activation seen in a control experiment using a virus in which OVAI was inserted into the stem of the viral NA protein (NA-SIIN) (Figure 4D, middle panels) (Bottermann et al., 2018). Again, noo upregulation was observed during mock infection. Taken together, our data with both the PB1-UFO(SIIN) and the NS-SIIN viruses indicate that, unless blocked by stop codons, uvORFs are translated and expressed during infection, and T cell immunosurveillance extends to peptides encoded by uvORFs.

Next, to probe if the expression of chimeric host-viral proteins has an impact on viral pathogenesis, we generated a battery of recombinant viruses, in which specific N-terminal extensions or uvORFs were knocked out through the introduction of premature stop codons (NP-Δext and UFO^Δ^, respectively). The viruses were generated either in the PR8 (Figures 4E, 4F, and S4D), A/WSN/33(H1N1) (WSN) (Figure S4E), or mouse-adapted A/California/04/2009(H1N1) (Cal09) (Figure S4F) backgrounds. We also generated the reciprocal control viruses carrying synonymous mutations (NP^SYN^;UFO^SYN^). Both genomic configurations of control and knockout viruses maintained intact the canonical viral ORFs (Table S3).

The mutant viruses did not display gross alterations in viral growth *in vitro* (Figures S4D–S4F). This was independent of viral background and also of the cell type infected (Figures S4D–S4F). To determine if interrupting upstream translation had effects *in vivo*, we focused on the NP-Δext and PB1-UFO^Δ^ viruses in the PR8 background. The strategy used to generate these viruses is shown in the top panels of Figures 4E and 4F.

We found that the NP-Δext viruses were less virulent in mice compared to the control NP-SYN viruses (Figure 4E), suggesting that NP-ext expression contributes to virulence. A similar role for NP-ext was recently proposed for the pandemic 2019 IAV (pdm2009) strain, in which an extended NP protein was found to contribute to virulence in mice and pigs (Wise et al., 2019). Importantly, however, pdm2009 viruses translate NP-ext from a uAUG encoded in the 5′ UTR of NP, but no corresponding uAUG is encoded by the PR8 virus used in our study.

The PB1-UFO^Δ^ viruses displayed increased virulence when compared to the PB1-UFO^SYN^ viruses *in vivo*, although in this case an effect was only observed at high infectious doses (Figure 4F). Gene expression analyses suggested that there were distinct transcriptomic signatures in the lungs of mice infected with high doses of the PB1-UFO^Δ^ or PB1-UFO^SYN^ viruses (Figures S4G and S4H; Table S4A). Gene Ontology analysis of differentially expressed genes indicated changes in a number of pathways, including leukocyte activation and pro-inflammatory cytokine secretion (Figure S4I; Table S5). Immune cell dysregulation may therefore be at least partially responsible for the differences in morbidity and mortality during infection with the PB1-UFO^Δ^ or PB1-UFO^SYN^ viruses.

Together, these functional data show that uvORFs are expressed during IAV infections, can be detected by the adaptive immune system, and can modulate the severity of infection.

### Chimeric Host-IAV Proteins Are Conserved

**Figure S5:**
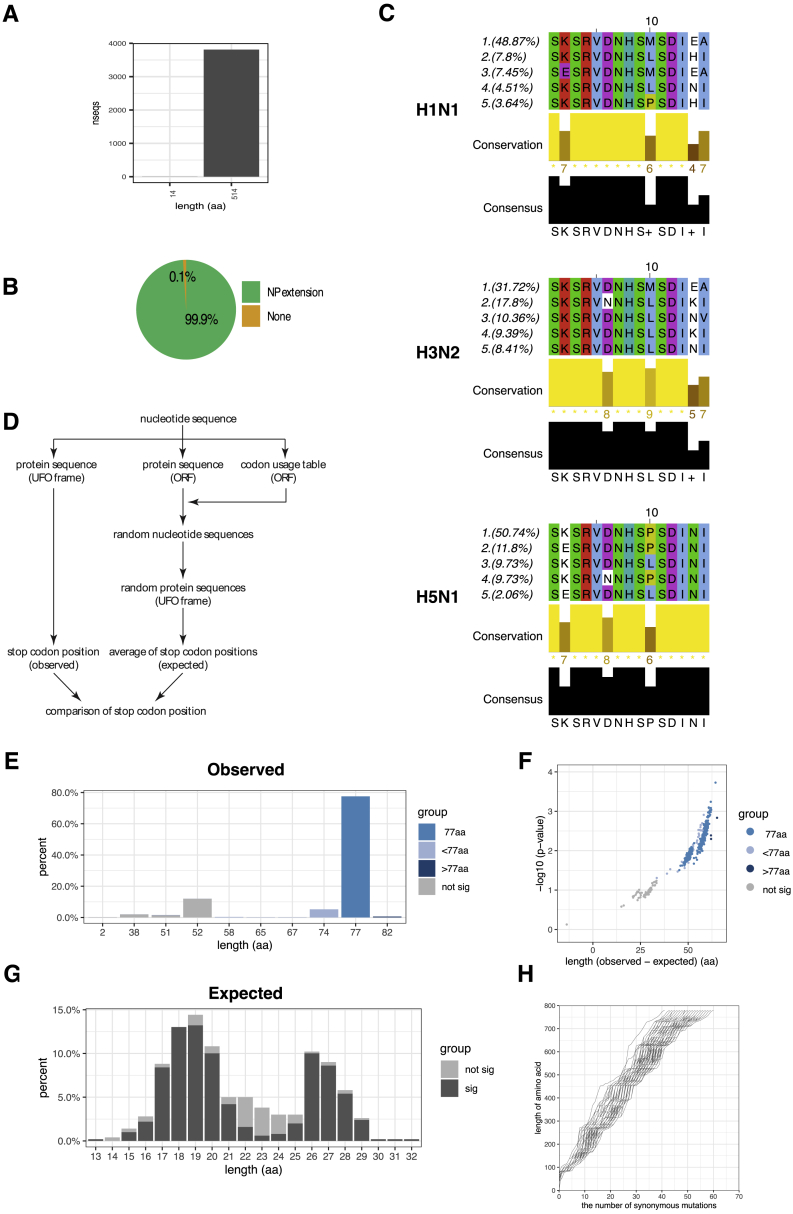
uvORFs Are Conserved, Related to Figure 5 (A) Bar plot showing the number of unique NP sequences that give rise to the full length, extended NP protein of ∼514aa, or those that result in truncated (non-extended) uvORFs. (B) Percentages of unique NP sequences that preserve the propensity to code for NP-extension. (C) Top five most common NP extension protein sequences in three types of influenza A strains, H1N1, H3N2 and H5N1. (D) Schematic showing the model used to calculate the expected versus observed PB1-UFO sequence lengths. (E) Density plot of predicted length of H3N2 PB1-UFO protein sequences. Sequences predicted to generate a protein of 77aa are shown in medium blue, shorter than 77aa in light blue, and those longer than 77aa are in dark blue. Sequences predicted not to generate PB1-UFO protein are shown in gray. (F) P value distribution/volcano plot of H3N2 PB1-UFO protein sequence length. Each dot represents the difference between observed length and expected length of each individual sequence. (G) Density plot showing the distribution of expected lengths of H3N2 PB1-UFO proteins, based on random codon-shuffled sequences. (H) Line plot showing the number of synonymous mutations in frame of WT H3N2 PB1 (x axis) that are required to generate stop codons in frame of H3N2 PB1-UFO (y axis).

We next asked if NP-ext and PB1-UFO are conserved across different strains. The ability to express NP-ext without interruption by stop codons in the 5′ UTR was maintained in 99.9% of IAV isolates present in the NCBI Influenza database (Zhang et al., 2017) (Figure S2, Figure S5A, and S5B). Sequence analysis of the translated 5′ UTR also suggested that N-terminally extended sequences would be similar within IAV subtypes (Figure S5C). There are many reasons why these sequences are conserved, including constraints imposed by RNA structure and the requirement to interact with the viral polymerase complex (Fodor, 2013). Whatever the primary selective pressure, the result of the conservation of the 5′ UTR sequence is that the ability to express NP-ext is nearly universal among IAV strains.

**Figure 5:**
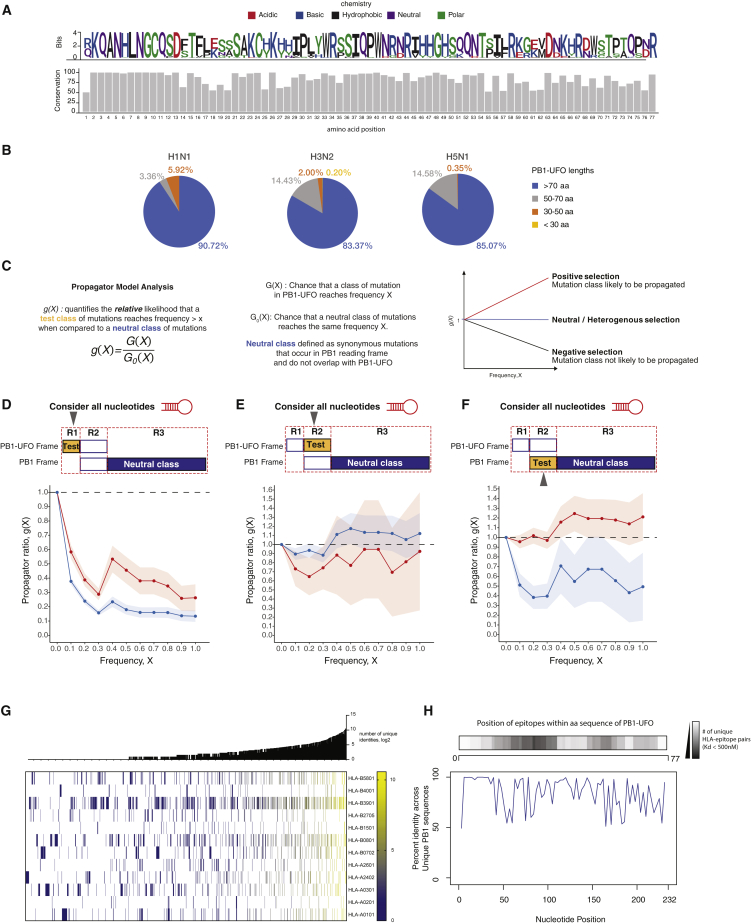
uvORFs Are Conserved (A) Conservation analysis of PB1-UFO protein sequences across all IAV subtypes. (B) Pie charts showing percentages of sequences in H1N1, H3N2, and H5N1 IAV subtypes that have a PB1-UFO that is 77 aa long (blue), 50–77 aa long (gray), 30–50 aa long (orange), and <30 aa long (yellow). (C) Outline of the propagator model analysis. Diagrams describe possible outcomes and interpretations of calculated *g*(*x*) ratios (D) Frequency propagator ratios of the indicated classes of mutations occurring in PB1-UFO relative to the PB1 open reading frame of H3N2 viruses. Top: regions used for the test (*G*(*x*); yellow), and neutral class (*G*_0_(*X*); blue) ratios are shown. The test class is the region of PB1-UFO ORF that overlaps only with the viral 5′ UTR; the neutral class consists of synonymous mutations in the PB1 ORF that do not overlap with PB1-UFO. All nucleotides positions were considered. Error bars indicate sampling uncertainties. See also Figure 5C for interpretations (E) Frequency propagator ratios, as in (D), but with the test class comprising the C-terminal region of the PB1-UFO ORF. (F) Frequency propagator ratios, as in (D), but with the test class comprising the region in the main PB1 ORF overlapping the PB1-UFO reading frame. (G) Number of predicted PB1-UFO epitope-allele interactions for frequent 11 human HLA alleles. Heatmaps show number of PB1-UFO epitopes derived from all possible unique identities and predicted to bind selected MHC-I alleles. Number of unique identities (i.e., unique influenza A virus sequences) encoding predicted epitopes are shown in histograms, next to the heatmaps. (H) Locations of PB1-UFO peptides that are predicted to result in strong (K_d_ <500 nM) unique interacting HLA-epitope pairs across the PB1-UFO reading frame. This plot is juxtaposed with percent identity plot of PB1-UFO (lower panel) across 3,140 unique PB1-UFO sequences taken from the NCBI Influenza Database (Zhang et al., 2017).

The ability to express PB1-UFO requires not only a lack of stop codons in the appropriate frame of the 5′ UTR, but also the maintenance of a uvORF overprinted on the canonical PB1 ORF. We first analyzed sequences of the IAV subtypes H1N1, H3N2, and H5N1. We found that PB1-UFO is conserved within each of these three virus subtypes (Figure 5A), and stop codons resulting in PB1-UFO proteins <77 aa long were infrequent (Figure 5B).

To understand the factors that contribute to the maintenance of PB1-UFO ORF length and amino acid sequence composition within the IAV, we first looked at the probability that an ORF similar in length to PB1-UFO could have arisen stochastically in the IAV PB1 segment. We used a sequence randomization model (Figure S5D) on the H3N2 subtype of IAV, the subtype for which the greatest number of complete sequences were available. We found that ∼77% of the sequences in the NCBI Influenza database (Zhang et al., 2017) encoded a 77-aa PB1-UFO (Figure S5E) that is significantly longer than the ∼15–30 aa long ORFs expected by chance (Figures S5E–S5G). We also found that these predicted ORFs would require multiple (30–70) additional synonymous mutations in order to generate an ORF that is of similar length to PB1-UFO (Figure S5H).

**Figure S6:**
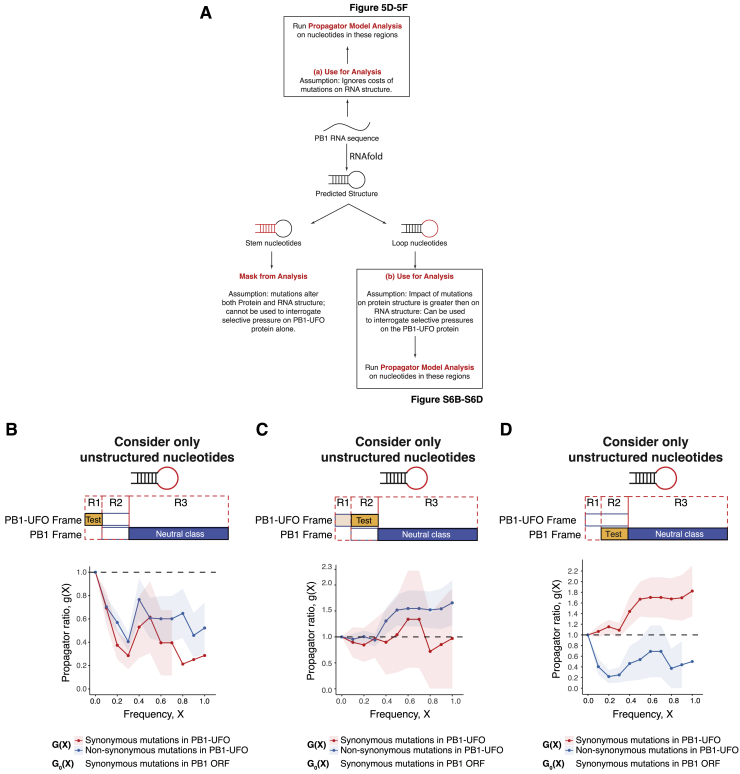
Controls Related to Propagator Analysis, Related to Figures 5C–5F (A) Schematic of analysis steps taken to quantify selection occurring on synonymous and non-synonymous mutations in the PB1-UFO ORF. Propagator model analyses were done by either not taking (Figure 5B and 5D) or taking the RNA structure of IAV PB1 segment into account (Figures 5C–5E). (B) Frequency propagator ratios of the indicated classes of mutations occurring in PB1-UFO relative to the PB1 open reading frame of H3N2 viruses. The region used to calculate the test class ratio (G(X)) is indicated in yellow, and the region used to calculate the neutral class ratio (G_0_(X)) is indicated in blue in the top schematic. Here, the test class is the region of the PB1-UFO ORF that overlaps only with the virally-encoded 5′UTR; the neutral class consists of synonymous mutations in the PB1 ORF that do not overlap with PB1-UFO. Only nucleotides within predicted loop regions (i.e., non-pairing) positions were considered. Error bars indicate sampling uncertainties. g(x)<1: negative selection, g(x)≈1: weak/heterogeneous selection; g(x)>1: positive selection; see also Figure 5C) (C) Frequency propagator ratios, as in (B), but with the test class comprising the C-terminal region of the PB1-UFO ORF. (D) Frequency propagator ratios, as in (B), but with the test class comprising the region in the main PB1 ORF overlapping the PB1-UFO reading frame.

The above analysis does not take into account constraints imposed by nucleotide biases in the viral UTR or canonical PB1 ORF or from viral RNA structure. To examine their roles in the maintenance of the PB1-UFO ORF we used the frequency propagator method (Luksza and Lässig, 2014, Strelkowa and Lässig, 2012) (Figures 5C and S6A). This method can determine if these factors imposed constraints on the PB1-UFO amino acid sequence. The model and its possible outcomes are shown and discussed in detail in Figures 5C and S6A and the STAR Methods.

Briefly, mutations that occur in the viral UTR region, which encodes the N-terminal part of PB1-UFO, undergo negative selection (Figure 5D; *g* < 1). This indicates that mutations in the viral UTR, should they occur, have a low probability of being propagated down the IAV strain tree. On the other hand, when we consider the nucleotide sequences that encode the overlapping regions of PB1-UFO and the canonical PB1 ORF, we see that there is heterogeneous/neutral selection occurring on mutations in the PB1-UFO ORF (*g* ≈ 1). This is most likely shaped by the requirement to maintain the main PB1 ORF sequence, as mutations that maintain the PB1 ORF aa sequence (synonymous mutations in PB1 ORF) are more likely to be fixed in the population (Figure 5E; red line; *g* < 1). Mutations that change the PB1 amino acid sequence instead undergo negative selection (Figure 5F; blue line; *g* < 1) and are unlikely to be propagated down the strain tree, consistent with PB1 ORF being fixed and essential for IAV.

Selection in these regions is unlikely to be dominated by RNA structural constraints because similar effects are observed when RNA secondary structure is taken into account for our analysis (Figures S6B–S6D). Overall, our analyses suggest that PB1-UFO conservation is largely dictated by the need to preserve both the viral UTR nucleotide sequence and the amino acid sequence of the main PB1 ORF. Taken together, this suggests that the evolution of the PB1-UFO ORF is heavily constrained by converging selective pressures.

Because we had shown that peptides derived from PB1-UFO could be presented to the immune system (Figures 4C and 4D), we asked whether epitope-HLA class I interactions could play a role in shaping PB1-UFO sequence. We found that multiple unique PB1-UFO peptides were predicted to bind to and interact with various HLA types (Figure 5G; Table S6). Notably, high-affinity (<500 nM) HLA-epitope pairs were concentrated in regions of PB1-UFO where conservation was low, suggesting that immune pressure on PB1-UFO may lead to some diversifying selection on the protein (Figure 5H).

### Chimeric Host-Virus Proteins of Other Viruses

**Figure S7:**
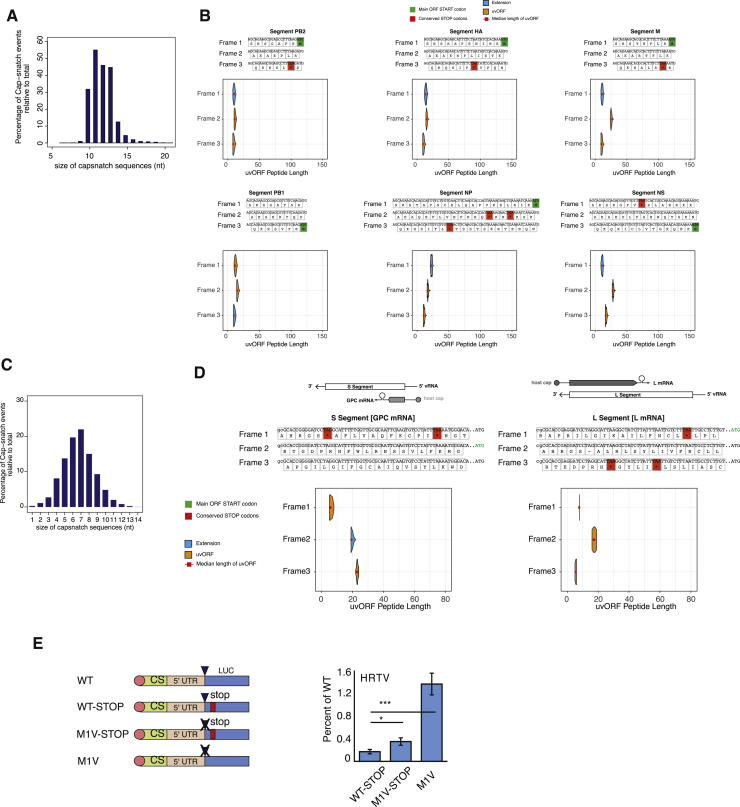
DEFEND-Seq and CAGE Analysis of Other Cap-Snatching Viruses, Related to Figure 6 (A) Distribution of lengths for cap-snatched sequences found in IBV, as determined by DEFEND-seq. (B) Host derived uAUGs give rise to long uvORFs (> 30aa). (Upper panels) Predicted peptide sequences derived upon translation of all three ribosome reading frames in the indicated IBV genome segments. (Lower panels) Predicted distribution of the lengths of new ORF and extension peptides generated from each reading frame of the viral 5′UTR. Peptide lengths are calculated based on AUG positions obtained through DEFEND-sequencing. (C) Distribution of lengths for cap-snatched sequences found in LASV infected cells, as determined by CAGE-seq. (D) Host derived uAUGs enable reverse sense genome segments of Lassa virus L and S to give rise to uvORFs and extensions. (Upper panels) Schematic of proteins encoded in the indicated reading frames in either the L or S segment. Lassa virus RNA is ambisense. (Middle panels) Predicted peptide sequences derived upon translation of all three reading frames in the reverse sense L and S segments. (Lower panels) Predicted distribution of the lengths of new ORFs and extension peptides generated from each reading frame of the viral 5′UTR. Peptide lengths are calculated based on AUG positions obtained through CAGE. (E) (Left panels) Schematic showing (in coding sense) the 5′ termini of viral reporter RNAs, in which a viral untranslated region (UTR) flanks a luciferase (Luc) reporter gene. Reporter RNAs were used to assess upstream translation in the mRNAs of Heartland virus (HRTV). The 5′ terminus of the mRNAs consisted of cap-snatched sequence from host mRNAs (cap), followed by a viral 5′ UTR (5′ UTR) and the reporter gene (Luc). Cap structures are indicated as circles, the most N-terminal AUG as a triangle, AUG mutations as crosses and stop codons as lines. (Right panels) Luc expression when these reporters were included in minireplicon assays, as a percentage of expression with the WT construct, showing the means and s.d. of 3 repeats compared to WT-STOP by Student’s 2-tailed t test (n.s.: p ≥ 0.05, ^∗^p < 0.05, ^∗∗∗^p ≤ 0.0005).

**Figure 6:**
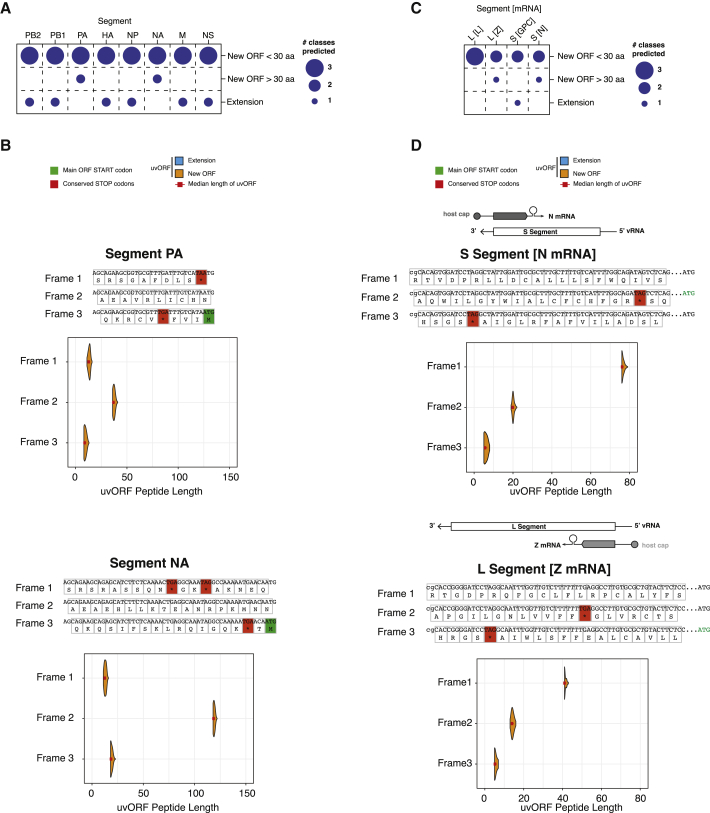
uvORFs Are Encoded by Cap-Snatching Viruses from Diverse Families (A) The number of host-virus chimeric protein species potentially encoded by influenza B virus (IBV; B/Wisconsin/01/2010). (B) Sequence analysis of the PA and NA segments of IBV, showing the translation of the 5′ UTR in all three reading frames and the predicted length distributions of N-terminal extensions to the main ORF and of overprinted new ORFs, calculated from uAUG positions in DEFEND-seq data. (C) The number of host-virus chimeric protein species potentially encoded by the ambisense genome of Lassa virus (LASV; Josiah strain), in both forward and reverse senses. The ORF encoded by the segment is indicated in the square brackets. (D) Sequence analysis of L and S segments of LASV in the indicated orientations, showing a schematic of genome organization, the translation of the 5′ UTR in all three reading frames, and the predicted length distributions of overprinted new ORFs, calculated from uAUG positions in CAGE-seq data.

Finally, we asked whether our finding that start-snatching generates novel ORFs could be generalized from IAV to other sNSVs. We began by looking at another member of the *Orthomyxoviridae* family, influenza B virus (IBV), by performing DEFEND-seq on A549 cells infected with IBV. The host-derived sequences that IBV obtains by cap-snatching had comparable median lengths to those appropriated by IAV (Figure S7A). Sequence analysis indicates that uAUG-initiated translation could read through the 5′ UTR of every IBV genome segment in at least one frame and predicted at least two long overprinted new ORFs (PA and NA segments) (Figures 6A and 6B), as well as N-terminal extensions of six of the eight major viral proteins (Figures 6A and S7B).

Next, we looked at other families of sNSVs. We performed CAGE analysis on cells infected by Lassa virus (LASV), a member of the family *Arenaviridae* and an emerging virus that in the past decade has caused several epidemics of hemorrhagic fever. LASV genomes comprise two ambisense segments. The median cap-snatched length of LASV mRNAs was seven nucleotides (Figure S7C) in agreement with structural prediction of the LASV polymerase (Wallat et al., 2014). Sequence analysis indicates that these uAUGs could lead to the translation of N-terminal extensions of the GPC protein, as well as the formation of two overprinted new ORFs of ∼50 and 80 aa from the viral mRNAs encoding the nucleoprotein (N) and Z proteins of LASV (Figure 6C, 6D, and S7D). The proportions of uAUGs detected in cap-snatched sequences from IBV and LASV were dependent on viral segments and ranged between 4% and 12% (Table S7).

We also tested the hypothesis that translation of UTR-derived sequences could occur in other sNSVs by using minireplicon assays encoding a luciferase reporter to a member of the *Phenuiviridae* (Heartland banyangvirus; L segment UTRs). By mutating the canonical AUG, we identified low but readily detectable levels of upstream translation (Figure S7E).

**Figure 7:**
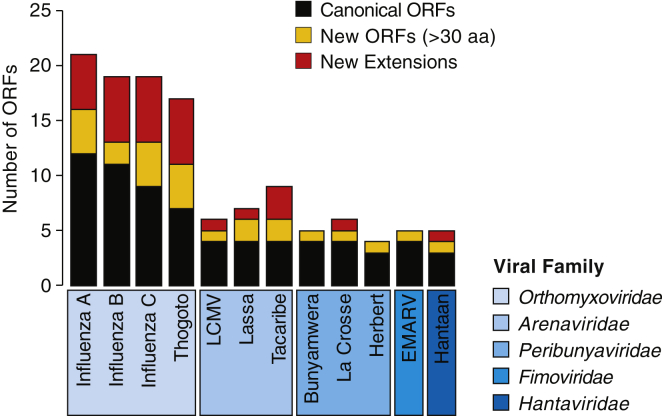
Start-Snatching Increases the Number of Potential ORFs in sNSVs The increase in number of potential ORFs in cap-snatching viruses when uvORFs are considered. Black, number of canonical ORFs; yellow, number of new overprinted ORFs >30 aa; red, number of new extensions. LCMV, lymphocytic choriomeningitis virus; EMARV, European mountain ash ringspot-associated emaravirus.

Overall, these data suggest that generation of chimeric virus-host ORFs is a common feature of sNSVs. To quantify the potential pervasiveness of this mechanism and the likelihood of novel ORFs being conserved and functionalized into new genes, we analyzed RNA virus genomic sequences for their propensity to generate novel proteins by performing *in silico* analyses of their genomes. Although the exact levels of upstream translation will depend on a range of factors, including the intrinsic properties of viral polymerase complexes and, potentially, mechanisms that modulate upstream AUG translation, our results indicate the genomic potential of start-snatching (Figure 7). Given that viral mRNA and proteins are among the most highly expressed biotypes in infected cells, our data support the idea that all cap-snatching virus could expand their proteome by start-snatching uAUGs from their hosts.

## Discussion

In this manuscript, we describe the existence of a mechanism employed by sNSVs to generate chimeric host-virus genes. This mechanism, “start-snatching,” involves the co-opting of start codons from host mRNA sequences to expand the viral proteome. This mechanism appears to be accessible to all sNSVs, including major human pathogens such as IAV and LASV. Start-snatching allows the translation of proteins from cryptic uvORFs, either as canonical viral proteins with N-terminal extensions, or as UFO proteins overprinted on the canonical viral ORF. In this study, we have identified examples of both types of uvORF in IAV infections. We have shown that translation can initiate on uAUGs in the host-derived sequence of viral mRNAs, and that this leads to the expression of chimeric host-virus proteins that can be detected in infected cells. In our hands, the ablation of uvORFs did not impact viral replication *in vitro* but had a moderate effect *in vivo*, which would be consistent with uvORFs encoding accessory proteins. We found that uvORFs can be recognized by the immune system, and we modeled the contribution of different evolutionary forces at play on uvORFs by characterizing viral-intrinsic and host-immune features that contribute to their evolution. Finally, we showed experimentally and by sequence analysis that the capability to express uvORFs through start-snatching is widespread among the sNSVs.

### Chimeric mRNAs Encode Novel Viral Proteins

We hypothesized that cap-snatching of sNSVs could generate ORFs that are encoded by two genomes (human and virus). Consistent with this, our analyses indicate that roughly 10% of IAV mRNA contains host-derived uAUGs (Figures 1D and S1B). Furthermore, uvORFs are translated in at least three of the eight IAV genome segments, generating NP-ext, PB2-UFO, and PB1-UFO (Figures 2 and 4). Genetic evidence suggested that many other uvORF proteins are also likely to be expressed, although we did not detect them in our current study, potentially due to the substantial sequence overlap of N-terminal extensions with canonical viral proteins and the short lengths of many overprinted ORFs. Overall, our analysis indicates that multiple families of viruses can generate chimeric RNAs and could produce proteins via this mechanism (Figures 6 and 7).

### Conservation and Function of uvORFs

Our analysis shows that most sNSV infections lead to expression of chimeric genes and uvORFs. Because they are host and virally encoded, it is therefore reasonable to ask who benefits from their expression. Key considerations in this regard, and based on our analysis are:

(1) Epitopes encoded in uvORFs are recognized by the adaptive immune system. MHC I presentation of uvORF-derived peptides poses the risk of an adaptive immune response against cells infected with sNSVs, analogous to the risks posed to IAV by the presentation of alternative reading frames (ARFs) and defective ribosomal products (DRiPs) (Dolan et al., 2010, Wei et al., 2019, Wei and Yewdell, 2017, Wei and Yewdell, 2019, Zanker et al., 2019). Indeed, the risks posed to the virus by the presentation of uvORFs are potentially even higher due to the high conservation of these sequences.
(2) Two uvORFs considered here (NP-ext and PB1-UFO) are both highly conserved across multiple strains of IAV. However, merely assessing conservation is insufficient, as other forms of selection also act on IAV genome sequences. In particular, genome packaging signals in the primary RNA sequence are concentrated in the terminal regions of each genome segment (Dadonaite et al., 2019, Gog et al., 2007, Hutchinson et al., 2010), resulting in a suppression of synonymous codon usage (Gog et al., 2007, Jagger et al., 2012). In overprinted regions, like PB1-UFO, there is also selective pressure conferred by the sequence encoding the canonical ORF. We observe both of these effects (Figures 5D–5F). Despite this, we also observe that (1) nonsense mutations do not occur frequently in the population (Figures S5D–S5H), and (2) missense mutations that do eventually accumulate in the PB1-UFO ORF tend to be those that change potentially immunogenic epitopes (Figures 5G and 5H).

This information, and the mere fact that full-length PB1-UFO is present in more than 75% of all IAV isolates and NP extensions are present in more than 99% of IAV isolates, suggests that multiple forces at the host-virus interface drive the virus to maintain the full-length proteins in their sequences. The relative contributions of distinct evolutionary forces in maintaining these proteins are not yet clear.

An important point to be made about uvORFs is that conservation and/or expression does not equate to functionality. While some uvORFs might have gained functions, we predict others will exist as afunctional, evolutionary spandrels. Such uvORFs are stuck in a place where they have to be made but suffer too many external constraints to productively sample evolutionary space for functionalization. All things considered, we can fairly surmise that any cost the virus might incur through uvORFs being made is outweighed by the fitness benefits of maintaining a genetic architecture that allows for their expression. The awareness of uvORF existence, and their pervasiveness in the viral world, is thus critical for our understanding of viral biology, viral evolution, and host immune surveillance.

### Gene Origination through Overprinting and the Mis-naming of "UTRs"

Genetic overprinting typically occurs when a pre-existing reading frame acquires mutations that enable translation in alternative reading frames while maintaining the function of the ancestral frame. This is an important mechanism for the creation of new proteins, especially in the context of compact genomes (viral, prokaryotic, and eukaryotic organelles) with little coding capacity (Keese and Gibbs, 1992, Kovacs et al., 2010, Poulin et al., 2003, Sabath et al., 2012).

While genetic overprinting could be selectively advantageous for some organisms, the evolution of overprinted genes is problematic. Any evolution of the overprinted ORF will be constrained by the effects of mutations in the underlying ORF. In addition, established overlapping ORFs typically have dedicated mechanisms for their expression, such as ribosomal scanning or frameshifting, which allow for efficient and regulated expression patterns. Exploring the limited evolutionary space that satisfies all of these constraints presumably requires the overprinted gene to provide a strong selective advantage.

Start-snatching exposes the 5′ coding regions of sNSV genomes to low levels of non-specific out-of-frame translation. This “genetic feature” could facilitate the evolution of novel genes through genetic overprinting, without having to evolve a dedicated method to express an overprinted ORF before that ORF could provide a selective advantage.

A similar argument applies to the evolution of alternative upstream translation mechanisms for N-terminally extended proteins: if an N-terminal extension provided by start-snatching was selectively advantageous, the virus could evolve to directly encode an uAUG in the UTR and make the generation of extended protein host-independent and heritable. In this respect, it is interesting to note that some recent strains of IAV have evolved to encode a uAUG in the UTR of NP that allows it to express an N-terminally extended protein that can modulate virulence (Wise et al., 2019). In essence, start-snatching might simply be a way to increase the chances of UTR translation by outsourcing uAUG to non-viral genomic material.

The translation of 5′ UTRs (that implies their misnaming) occurs frequently in eukaryotic genes. uORFs are, in fact, pervasively expressed, with some functioning as short biologically active polypeptides (Andrews and Rothnagel, 2014, Calvo et al., 2009, Combier et al., 2008, Sendoel et al., 2017, Starck et al., 2016, Wang and Rothnagel, 2004, Wen et al., 2009). uORFs are abundantly expressed in cancer cells (Sendoel et al., 2017) and activated T cells (Starck et al., 2016). Overall, future work will be needed to redefine what, in reality, a gene is.

### Lessons for Other Viruses

The capacity of a pathogen to overcome host barriers and establish infection is based on the expression of pathogen-derived proteins. To understand how a pathogen antagonizes the host and establishes infection we need to have a clear understanding of what proteins a pathogen encodes, how they function, and the manner in which they contribute to virulence. The current dogma about many life-threatening pathogens is that they encode a small repertoire of proteins because of their limited genome size. RNA viruses, such as IAV, are a prime example of this. Here, we have shown that IAV, IBV, LASV, and likely most, if not all, other sNSVs, can use host RNA to expand their genetic repertoire. Similar to novel human genes which originated from other mechanisms and contributed to organismal evolution (Kaessmann, 2010, Ohno, 1970), we expect chimeric genes to shape (and have shaped) host-virus relationships.

## STAR★Methods

### Key Resources Table

**Table:** 

REAGENT or RESOURCE	SOURCE	IDENTIFIER
**Antibodies**		

anti-CD25-APC (PC61.5)	Thermo Fisher	Cat #17-0251-82; RRID: AB_469366
anti-CD8-Alexaflor488 (53-6.7)	Thermo Fisher	Cat #53-0081-82; RRID: AB_469897
anti-Vα2-E450 (B20.1)	Thermo Fisher	Cat #48-5812-82; RRID: AB_10804752
anti-Vβ5-PE (MR9-4)	BD Biosciences	Cat # 553190; RRID: AB_394698
anti-CD44-PerCp-Cyanine5.5 (IM7)	Thermo Fisher	Cat #45-0441-82; RRID: AB_925746
anti-CD69-PE-Cy7 (H1.2F3)	Thermo Fisher	Cat #25-0691-82; RRID: AB_469637
Anti-NP antibody	BioRad	Cat # MCA400; RRID: AB_2151884
m-IgGκ BP-HRP	Santa Cruz	Cat # sc-516102; RRID: AB_2687626

**Bacterial and Virus Strains**		

A/Puerto Rico/8/34 (H1N1) (PR8)	de Wit et al., 2004	N/A
PR8; PB1-UFO^Δ^	This study	N/A
PR8; PB1-UFO^SYN^	This study	N/A
PR8; PB1-EXT+	This study	N/A
PR8; NP-EXT^Δ^	This study	N/A
PR8; NP-EXT^SYN^	This study	N/A
A/California/04/09(H1N1) (Cal09)	Ye et al., 2010	N/A
Cal09; PB1-UFO^Δ^	This study	N/A
Cal09; PB1-UFO^SYN^	This study	N/A
Cal09; PB2-UFO^Δ^	This study	N/A
Cal09; PB2-UFO^SYN^	This study	NA
Cal09; PA-UFO^Δ^	This study	NA
Cal09; PB1-UFO^SYN^	This study	NA
Cal09; HA-UFO^Δ^	This study	NA
Cal09; HA-UFO^SYN^	This study	NA
A/WSN/33(H1N1) (WSN)	Hoffmann et al., 2000	N/A
WSN; PB1-UFO^Δ^	This study	N/A
WSN; PB1-UFO^SYN^	This study	N/A
WSN; PB2-UFO^Δ^	This study	N/A
WSN; PB2-UFO^SYN^	This study	N/A
WSN; PA-UFO^Δ^	This study	N/A
WSN; PA-UFO^SYN^	This study	N/A
WSN; HA-UFO^Δ^	This study	N/A
WSN; HA-UFO^SYN^	This study	N/A
PB1-UFO(SIIN)	This study	N/A
NS-UFO(SIIN)	This study	N/A
PB1-SIIN	Wei et al., 2019	N/A
NA-SIIN	MRC-University of Glasgow Centre for Virus Research; As Bottermann et al., 2018	N/A
A/Udorn/72(H3N2)	The Roslin Institute, University of Edinburgh; As Clohisey et al., 2020	N/A
LASV (Josiah strain)	Department of Pathology, the University of Texas Medical Branch	N/A
B/Wisconsin/01/2010	Department of Microbiology, Icahn School of Medicine at Mount Sinai	N/A

**Biological Samples**		

Primary CD14+ human monocytes	The Roslin Institute, University of Edinburgh; As Clohisey et al., 2020	N/A

**Chemicals, Peptides, and Recombinant Proteins**		

Dulbecco’s Modified Eagle Medium (DMEM)	Thermo Fisher / GIBCO	Cat#11965175
Minimum Essential Medium (MEM)	Sigma-Aldrich	Cat# 51411C
Purified Agar	Oxoid	Cat #: LP0028
Trypsin from bovine pancreas, TPCK-treated	Sigma-Aldrich	Cat #: T1426-500MG
Protease Inhibitor Cocktail Set III, EDTA-Free - Calbiochem	EMD Millipore	Cat# 539134-10ML
Trypsin	Sigma-Aldrich	Cat# T8802-100MG
TRIzol Reagent	Thermo Fisher Scientific	Cat#15596018
SimplyBlueTM SafeStain	Thermo Fisher Scientific	Cat# LC6060
NuPage 4−12% BT Gel 1.5mm 12w 10 Per Box	Thermo Fisher Scientific	Cat# NP0322BOX
MG-132	Sigma-Aldrich	Cat# M7449-1ML
NuPAGE MOPS SDS Running Buffer (20X)	Thermo Fisher Scientific	Cat# NP0001
Ovalbumin (257-264) chicken	Sigma-Aldrich	Cat# S7951
LT-1 transfection reagent	Mirius	Cat# MIR 2304
recombinant human colony-stimulating factor 1	A gift from Chiron, Emeryville, CA, US; As Clohisey et al., 2020	N/A
Lys-C lysyl endopeptidase	Wako	121-05063
Harringtonine	LKT biochemicals	H0169
Cycloheximide	Sigma-Aldrich	Cat# C7698
Sequencing grade modified trypsin	Promega	9PIV511

**Critical Commercial Assays**		

Dual Luciferase Reporter Assay System	Promega	Cat#E1910
CD8a+ T Cell Isolation Kit	Miltenyi Biotec	Cat#130-104-075
EasySep Mouse CD8+ T Cell Isolation Kit	StemCell Technologies	Cat# 19853
PureLink RNA Mini Kit 250 Reactions	Thermo Fisher Scientific	Cat# 12183025
PureLink DNase Set	Thermo Fisher Scientific	Cat# 12185010
miRNeasy Mini Kit	QIAGEN	Cat# 217004
Q5 site directed mutagenesis kit	NEB	Cat# E0554S
Ribo-Zero Gold rRNA Removal Kit (Human/Mouse/Rat)	Illumina	Cat# MRZG12324
SMARTer total RNA Pico kit	Clontech	Cat# 634411
TruSeq Stranded Total RNA Library Prep Kit	Illumina	Cat # 20020596

**Deposited Data**		

CAGE sequencing of WSN IAV virus infected cells	Clohisey et al., 2020	https://fantom.gsc.riken.jp/5/data/
DEFEND seq of PR8 IAV infected A549 cells	Rialdi et al., 2017	GEO: GSE96677
DEFEND seq of IBV infected A549 cells	This study	GEO: GSE85474
Ribosome Profiling of PR8 IAV infected cells	This study	GEO: GSE148245
CAGE sequencing of LASV infected vero cells	This study	GEO: GSE148122
RNA seq of PR8; PB1-UFO^Δ^ and PR8;PB1-UFO^SYN^ infected mouse lungs	This study	GEO: GSE128519
GISAID Database	Shu and McCauley, 2017	https://www.gisaid.org
NCBI Influenza Virus Database	Zhang et al., 2017	http://www.ncbi.nlm.nih.gov/genomes/FLU/Database
Mass spectrometry Data: PR8 IAV infected A549 and 293T cells	This study	Table S2A
Mass spectrometry Data: WSN IAV Virions	Hutchinson et al., 2014	https://massive.ucsd.edu/ProteoSAFe/datasets.jsp using the MassIVE ID MSV000078740; Table S2B
Mass spectrometry Data: Immunoprecipitation of PR8 IAV RdRp	Heaton et al., 2016	Table S2C

**Experimental Models: Cell Lines**		

Dog: MDCK	ATCC	CCL-34; RRID: CVCL_0422
Human: A549	ATCC	CCL-185; RRID: CVCL_0023
Human: 293T	ATCC	CRL-3216; RRID: CVCL_0063
Cow: MDBK	Sigma	90050801-1VL; RRID: CVCL_0421
Monkey: Vero	ATCC	CCL-81; RRID: CVCL_0059
Mouse: DC2.4	Sigma-Aldrich	Cat# SCC142; RRID: CVCL_J409
Hamster: BSR-T7/5	Buchholz et al., 1999	N/A

**Experimental Models: Organisms/Strains**		

Mouse: BALB/cJ (6-8 weeks)	Jackson Laboratories	00651
Chicken: Specific Pathogen Free Fertile Eggs	Charles River	Cat #: 10100329
Mouse: OT-I: C57BL/6-Tg(TcraTcrb)1100Mjb/J	The Jackson Laboratory / in-house; Hogquist et al., 1994	Cat# 003831; RRID: IMSR_JAX:003831
Mouse: C57BL/6 (10-14 weeks)	Envigo	N/A

**Oligonucleotides**		

DEFEND-seq cDNA synthesis–3’ primer	Rialdi et al., 2017	N/A
qPCR Primers	This Study	Table S4B

**Recombinant DNA**		

PR8 pDUAL plasmids	A kind gift of Prof Ron Fouchier; de Wit et al., 2004	N/A
Cal09 pDP2002 plasmids	A kind gift of Prof Daniel Perez.; Ye et al., 2010	N/A
pT7HRTMRen(-)	MRC-University of Glasgow Centre for Virus Research; Rezelj et al., 2019	N/A
pTMHRTN	MRC-University of Glasgow Centre for Virus Research; Rezelj et al., 2019	N/A
pTMHRTL	MRC-University of Glasgow Centre for Virus Research; Rezelj et al., 2019	N/A
pTM1-FFLuc	MRC-University of Glasgow Centre for Virus Research; Rezelj et al., 2019	N/A
pRL-TK	Promega	E2241

**Software and Algorithms**		

DESeq2	Love et al., 2014	https://bioconductor.org/packages/release/bioc/html/DESeq2.html
Bowtie	Langmead et al., 2009	http://bowtie-bio.sourceforge.net/index.shtml
MaxQuant	Cox and Mann, 2008	https://www.biochem.mpg.de/5111795/maxquant
Cutadapt	Martin, 2011	https://cutadapt.readthedocs.io/en/stable/
STAR	Dobin et al., 2013	https://github.com/alexdobin/STAR
FlowJo	Treestar	N/A
Metascape	Zhou et al., 2019	https://metascape.org/gp/index.html#/main/step1
Vienna RNA Webserver	Gruber et al., 2008	http://rna.tbi.univie.ac.at
FastTree	Price et al., 2010	http://www.microbesonline.org/fasttree
RAxML	Stamatakis, 2014	https://cme.h-its.org/exelixis/web/software/raxml/index.html
TreeTime	Sagulenko et al., 2018	https://github.com/neherlab/treetime
PANDASeq	Masella et al., 2012	https://github.com/neufeld/pandaseq
NetMHC (v3.4 and v4.0)	Andreatta and Nielsen, 2016	https://services.healthtech.dtu.dk/service.php?NetMHC-4.0
MUSCLE	Edgar, 2004	https://www.drive5.com/muscle/
HISAT2	Kim et al., 2015	http://daehwankimlab.github.io/hisat2
Prism 8	Graphpad	N/A

### Resource Availability

#### Lead Contact

Further information and requests for reagents may be directed to and will be fulfilled by Lead Contact Ivan Marazzi (ivan.marazzi@mssm.edu).

#### Materials Availability

All unique/stable reagents generated in this study are available from the Lead Contact with a completed Materials Transfer Agreement.

#### Data and Code Availability

The datasets for CAGE sequencing of A/Udorn/72 (H3N2) IAV virus infected cells are reported in Clohisey et al. (2020) deposited in https://fantom.gsc.riken.jp/5/data/. Datasets for DEFEND-seq of PR8-IAV infected A549 cells were taken from a pre-existing dataset [GEO: GSE96677] (Rialdi et al., 2017). DEFEND-seq of IBV infected cells were generated in this study and deposited in GEO: GSE85474. Ribosome profiling profile of PR8 IAV infected cells were generated in this study and deposited in GEO: GSE148245. The datasets for CAGE sequencing of LASV infected Vero cells were generated in this study and deposited in GEO: GSE148122. RNA seq of PR8; PB1-UFOΔ and PR8;PB1-UFOSYN infected mouse lungs was generated in this study and deposited in GSE128519. Mass spectrometry data for PR8 infected IAV infected A549 and 293 cells was generated in this study and presented in Table S2A. Mass spectrometry of WSN IAV virions was analyzed from datasets generated in Hutchinson et al. (2014), and taken from https://massive.ucsd.edu/ProteoSAFe/datasets.jsp using the MassIVE ID MSV000078740. Tables are also found in Table S2B. Mass spectrometry data for PB1-UFO interactions with IAV polymerase subunits was analyzed using datasets from Heaton et al. (2016) and presented in Table S2C.

### Experimental Model and Subject Details

#### Cells cultures

Madin–Darby Canine Kidney (MDCK) cells, A549 human lung epithelial cells, Vero (ATCC-CCL81) and 293T human embryonic kidney cells were cultured in Dulbecco’s Modified Eagle’s Medium (DMEM; GIBCO) supplemented with 10% fetal bovine serum (FBS; GIBCO). Madin-Darby Bovine Kidney (MDBK) cells were cultured in Minimum Essential Medium (MEM; Sigma) supplemented with 2 mM L-glutamine and 10% fetal calf serum (FCS). BSR-T7/5 golden hamster cells (Buchholz et al., 1999) were cultured in Glasgow Minimal Essential Medium (GMEM) supplemented with 10% FCS and 10% tryptose phosphate broth under G418 selection. All cells were maintained at 37ᵒC and 5% CO2.

#### Mice

For infection studies: Six to eight-week-old female BALB/c mice were obtained from Jackson Laboratories (Bar Harbor, ME). All mice infection procedures were performed following protocols approved by the Icahn School of Medicine at Mount Sinai Institutional Animal Care and Use Committee (IACUC). Animal studies were carried out in strict accordance with the recommendations in the Guide for the Care and Use of Laboratory Animals of the National Research Council.

For antigen presentation experiments: female OTI (Hogquist et al., 1994) mice were bred in-house on a mixed genetic background. Animals were kept in dedicated barrier facilities, proactive in environmental enrichment under the EU Directive 2010 and Animal (Scientific Procedures) Act (UK Home Office license number 70/8645) with ethical review approval (University of Glasgow). Animals were cared for by trained and licensed individuals and humanely sacrificed using Schedule 1 methods.

For BMDC Isolation: 10-14 week old naive female C57BL/6 mice, purchased from Envigo (UK) and maintained at the University of Glasgow under standard animal husbandry conditions in accordance with UK home office regulations and approved by the local ethics committee.

#### Virus Strains

##### Wild-type viruses

A/Puerto Rico/8/34(H1N1) (PR8) virus was generated by reverse genetics and propagated in 9-11 day old embryonated chicken eggs (Charles River, Cat # 10100329). Mouse-adapted A/California/04/09(H1N1) (Cal09) was generated by reverse genetics (Ye et al., 2010) and propagated on MDCK cells in the presence of 1 μg/ml TPCK-trypsin, as described previously (Hutchinson et al., 2008). The influenza virus A/WSN/33(H1N1) (WSN) (Hoffmann et al., 2000) was propagated on MDBK cells. A/Udorn/72(H3N2) (Udorn) was propagated on MDCK cells in the presence of 1 μg/ml TPCK-trypsin, as described previously (Clohisey et al., 2020, Hutchinson et al., 2014). Plaque assays were carried out in MDCK cells and visualized by immunocytochemistry or staining with crystal violet or Coomassie blue, as previously described (Gaush and Smith, 1968) (See below also for method details).

##### Mutant viruses

All mutant and control viruses were generated using a plasmid-based reverse genetics system (Fodor et al., 1999, Ye et al., 2010), using either the A/Puerto Rico/8/1934 (PR8), A/WSN/33 (WSN) or mouse-adapted A/California/4/09 (Cal09) strains as the backbone. Plasmids used for reverse genetics were the PR8 pDUAL plasmids (de Wit et al., 2004) and the Cal09 pDP2002 plasmids (Ye et al., 2010) (a kind gift of Prof Daniel R. Perez (University of Georgia, USA). Site-directed mutagenesis of plasmids was performed using the Q5 site-directed mutagenesis kit (QIAGEN); the edited NS segment sequence required for the PR8-NS.F3.SIIN mutant virus (described in Figure 4) was synthesized by Genewiz.

##### PB1-UFO(SIIN) virus

OVA257-264 (SIINFEKL) epitope was inserted into the 5′UTR of the PB1 segment of the influenza A virus (IAV) genome at position 1 before the PB1 start codon. This insertion did not result in an N-terminal extension of or mutations in the PB1 protein, but results in the insertion of the OVA257-264 antigenic epitope in frame with the PB1-UFO protein.

##### NS-UFO(SIIN) virus

The OVA257-264 (SIINFEKL) epitope was inserted into frame 2 of the NS segment of the IAV genome, in a region corresponding to the linker sequence of the NS1 protein (encoded in frame). This effectively replaced codons 79-84 of NS1, while retaining the sequence of NEP. The replacement sequence was flanked by two upstream nucleotides and one downstream nucleotide to introduce a frameshift into frame 2. Premature stop codons in frame 2 were also mutated at positions −4, 27, 32, 74 and 77, relative from the start codon of NS1, to generate a 106 amino acid long NS-UFO sequence, extending it from the original 4 amino acid long uvORF in reading frame 2.

##### PB1-SIIN virus and NA-SIIN viruses

These viruses have been described in Wei et al. (2019) and Bottermann et al. (2018) respectively.

##### PR8; PB1-UFO^Δ^, PR8; PB1-UFO^SYN^, PR8; PB1-EXT+ viruses

*PR8; PB1-UFO*^*Δ*^ contains a C to T nucleotide substitution 9 nucleotides after the start of PB1 open reading frame. This generates a premature stop codon in the PB1-UFO ORF. Its control virus, *PR8; PB1-UFO*^*SYN*^, contains a C to G nucleotide substitution at the same position. Both viruses retain the amino acid sequence of the PB1 ORF. *PR8; PB1-EXT+* contains a T to C nucleotide substitution three nucleotides before the start of PB1 open reading frame. This disrupts a conserved stop codon (“TGA”) in frame with PB1 ORF, resulting in the N-terminal extension of the PB1-ORF. PB1-UFO ORF is maintained in this virus. Mutations were confirmed by sequencing both plasmids and viruses. All viruses were expanded in 9-11 day old embryonated chicken eggs after rescue. The stock virus titers were calculated from the average of three independent experiments.

##### PR8; NP-EXT^Δ^, PR8; NP-EXT^SYN^ viruses

PR8; NP-EXT^*Δ*^ contains an A to T nucleotide substitution 6 nucleotides before the start of the NP open reading frame. This generates an in-frame stop codon that results in the loss of the N-terminal NP-extension. Its control virus, PR8; NP-EXT^SYN^, bears an A to G nucleotide substitution at the same position in the UTR, preserving the NP-extension. Mutations were confirmed by sequencing both plasmids and viruses, and 3 independent plaque purified clones of each virus, grown on MDCK cells, were used in subsequent experiments. Stock virus titers were calculated from the average of three independent experiments.

##### WSN; PB1-UFO^Δ^, WSN; PB1-UFO^SYN^, Cal09; PB1-UFO^Δ^, Cal09; PB1-UFO^SYN^ viruses

*WSN; PB1-UFO*^*Δ*^ and *Cal09; PB1-UFO*^*Δ*^ viruses contain C to U nucleotide substitutions 9 nucleotides after the start of PB1 open reading frame. This generates a premature stop codon in the PB1-UFO ORF. Their control viruses, WSN*; PB1-UFO*^*SYN*^ and *Cal09; PB1-UFO*^*SYN*^ respectively, contain C to G nucleotide substitutions at the same positions. All the viruses retain the amino acid sequence of the PB1 ORF.

##### WSN; PB2-UFO^Δ^, WSN; PB2-UFO^SYN^, Cal09; PB2-UFO^Δ^, Cal09; PB2-UFO^SYN^ viruses

*WSN; PB2-UFO*^*Δ*^ and *Cal09; PB2-UFO*^*Δ*^ viruses contain A to T nucleotide substitutions 12 nucleotides after the start of PB2 open reading frame. This generates a premature stop codon in the PB2-UFO ORF. Their control viruses, *WSN; PB2-UFO*^*SYN*^ and *Cal09; PB2-UFO*^*SYN*^ respectively, contain a A to C nucleotide substitutions at the same position. All the viruses retain the amino acid sequence of the PB2 ORF.

##### WSN; PA-UFO^Δ^, WSN; PA-UFO^SYN^, Cal09; PA-UFO^Δ^, Cal09; PA-UFO^SYN^ viruses

*WSN; PA-UFO*^*Δ*^ and *Cal09; PA-UFO*^*Δ*^ viruses contain C to T nucleotide substitutions 42 nucleotides after the start of PA open reading frame. This generates a premature stop codon in the PA-UFO ORF. Their control viruses, *WSN; PA-UFO*^*SYN*^ and *Cal09; PA-UFO*^*SYN*^ respectively, contain C to A nucleotide substitutions at the same position. All the viruses retain the amino acid sequence of the PA ORF.

##### WSN; HA-UFO^Δ^, WSN; HA-UFO^SYN^ viruses

*WSN; HA-UFO*^*Δ*^ viruses contain A to T nucleotide substitutions 45 nucleotides after the start of HA open reading frame. This generates a premature stop codon in the HA-UFO ORF. Their control viruses, WSN*; HA-UFO*^*SYN*^ and *Cal09; PA-UFO*^*SYN*^ respectively, contain A to C nucleotide substitutions at the same position. All the viruses retain the amino acid sequence of the HA ORF.

##### Cal09; HA-UFO^Δ^, Cal09; HA-UFO^SYN^ viruses

*Cal09; HA-UFO*^*Δ*^ viruses contain G to T nucleotide substitutions 52 nucleotides after the start of HA open reading frame. This generates a premature stop codon in the HA-UFO ORF. Its control virus, *Cal09; HA-UFO*^*SYN*^ contains an G to C nucleotide substitution at the same position. All the viruses retain the amino acid sequence of the HA ORF.

##### Primary CD14+ human monocytes

Primary CD14+ human monocytes were isolated from whole blood samples under ethical approval from Lothian Research Ethics Committee (11/AL/0168). Cells were obtained from blood donated by 4 anonymous healthy volunteers. Volunteers were not treated with any drugs. Some volunteers have donated blood used in multiple experiments outside this study. Health status is not assessed.

##### Plasmids

Plasmids used for HRTV minireplicon assays were the Renilla-luciferase-encoding pT7HRTMRen(–); the viral-gene-encoding pTMHRTN and pTMHRTL and the firefly-luciferase-encoding control plasmid pTM1-FFluc (Rezelj et al., 2019).

### Method Details

#### Growth kinetics of Viruses in Cell Culture

A549 or MDCK cells were infected with the indicated viruses at a multiplicity of infection (MOI) of 0.001 and incubated for one hour at 37°C. Infected cells were washed twice, and then cultured with Opti-MEM and TPCK-treated trypsin at 37°C for 72 h. Supernatants were collected at the indicated time points. Viral titers were determined by plaque assays.

#### Quantification of IAV titers by Plaque Assays

Plaque assay in MDCK cells were performed as described previously (Gaush and Smith, 1968). Briefly, serially diluted culture supernatants of infected cells were adsorbed on layers of confluent MDCK cells for 1 hour. Infected cells were then overlaid with 2ml of DMEM, 25mM HEPES, 2mM glutamine, 100ug/ml penicillin-streptomycin, 1ug/ml TPCK-trypsin and 0.8% Oxoid Agar. Plates were incubated for 48-72h until plaques were observed. Plaque were then fixed in 4% formaldehyde and visualized through staining with 1% crystal violet solution. Alternatively, MDCK cells were overlaid with DMEM mixed 1:1 with 2% (w/v) low gelling temperature agarose in PBS and supplemented with 1ug/ml TPCK-trypsin, incubated for 48-72h until plaques were observed, and then either fixed and stained directly (with 0.2% (w/v) Coomassie Brilliant Blue R in 7.5% (v/v) acetic acid and 50% (v/v) ethanol) or fixed in 80% chilled acetone and visualized by immunocytochemistry (permeabilized in 1% Triton X-100 in PBS, blocked in 10% FBS in PBS, immunostained with mouse anti-NP (BioRad: Cat# MCA400) and peroxidase-conjugated rabbit anti-mouse IgG (Santa Cruz; Cat # sc-516102) and visualized with True Blue Peroxidase).

#### Ribosome profiling and analysis

A549 cells were infected in a 10cm dish with A/Puerto Rico/8/1934 (H1N1, PR8) at a MOI of 3. At 8h post infection, ribosome profiling libraries were prepared as previously described (McGlincy and Ingolia, 2017) with the following exceptions. Infected cells were treated with either DMSO or 5μg/mL harringtonine for 15 minutes. Cell lysis was performed by flash freezing in liquid nitrogen prior to the addition of ice-cold lysis buffer. rRNA removal was performed as previously described (Wei et al., 2019). Sequencing was performed two lanes of a HiSeq using a 2x150 bp configuration.

#### Mass Spectrometry experiments (in infected cell lysates)

A549 or HEK293T cells were infected with PR8 virus stock at multiplicities of infection of 3 and 5 respectively. At 8h or 24h post infection, cells were scraped, washed twice in PBS with protease inhibitors (Calbiochem), before being snap-frozen in liquid nitrogen. Where indicated, MG132 was added to the cell culture media 4h prior to sample collection. Mock infected samples were included as negative controls. To prepare cell lysates for mass spectrometry, cell pellets were lysed in lysis buffer (50mM Tris pH8, 1% NP-40, 100mM NaCl, protease inhibitors) on ice. NaCl concentration was then brought up to 500mM by adding salt drop-wise into the solution while agitating. Lysates were rotated for 30min at 4°C before an equal volume of water was added to the sample to bring NaCl concentration back to 250mM. Samples were then centrifuged at full speed for 15 min at 4°C. 4x Laemmli buffer (200mM Tris-HCl pH6.8, 8% SDS, 40% glycerol, 0.588M B-mercaptoethanol, 50mM EDTA and 0.08% Bromophenol Blue) was then added to the supernatant to 1x concentration, and 5μl of the lysate was loaded on a 4%–12% Bis-Tris gel (Novex). Gels were run under a hood for 150V for 1h15min in 1X MOPS running buffer and stained in SimplyBlue™ SafeStain (Invitrogen), following the manufacturer’s recommended protocol. Once stained, gel bands corresponding to 40-60kDa and < 15kDa were excised. Gel slices were subject to in-gel tryptic digests as previously described (Rosenfeld et al., 1992).

Digested samples were analyzed on a Thermo Fisher Orbitrap Fusion mass spectrometry system equipped with an Easy nLC 1200 ultra-high pressure liquid chromatography system interfaced via a Nanospray Flex nanoelectrospray source. Samples were injected on a C18 reverse phase column (25 cm × 75 μm packed with ReprosilPur C18 AQ 1.9 μm particles). Peptides were separated by an organic gradient from 5% to 30% ACN in 0.1% formic acid over 70 minutes at a flow rate of 300 nL/min. The MS continuously acquired spectra in a data-dependent manner throughout the gradient, acquiring a full scan in the Orbitrap (at 120,000 resolution with an AGC target of 200,000 and a maximum injection time of 100 ms) followed by as many MS/MS scans as could be acquired on the most abundant ions in 3 s in the dual linear ion trap (rapid scan type with an intensity threshold of 5000, HCD collision energy of 29%, AGC target of 10,000, a maximum injection time of 35 ms, and an isolation width of 1.6 m/z). Singly and unassigned charge states were rejected. Dynamic exclusion was enabled with a repeat count of 1, an exclusion duration of 20 s, and an exclusion mass width of ± 10 ppm. Raw mass spectrometry data were assigned to human protein sequences and MS1 intensities extracted with the MaxQuant software package (version 1.6.8) (Cox and Mann, 2008). Data were searched against the SwissProt human protein database (downloaded on October 10, 2019) and a custom influenza A virus database comprising all six open-reading frames greater than 10 amino acids for the IAV (strain PR-8) genomic sequence. Variable modifications were allowed for N-terminal protein acetylation, methionine oxidation, and lysine acetylation. A static modification was indicated for carbamidomethyl cysteine. All other settings were left using MaxQuant default settings.

#### Mass Spectrometry experiments (in virions)

The purification of influenza virions and collection of mass spectra by LC-MS/MS has been described previously (Hutchinson et al., 2014), and followed previously-described protocols for purification, mass spectrometry and data analysis (Hutchinson and Stegmann, 2018). Briefly, the IAV WSN was propagated on MDBK cells. Six viral stocks were prepared, of which half were subjected to haemadsorption on chicken red blood cells to stringently remove non-viral material. Virus particles were then purified by sucrose gradient ultracentrifugation, lysed in urea, reduced, alkylated and digested with trypsin and LysC. Tryptic peptides were analyzed by liquid chromatography and tandem mass spectrometry (LC-MS/MS) using an Ultimate 3000 RSLCnano HPLC system (Dionex, Camberley, UK) run in direct injection mode and coupled to a Q Exactive mass spectrometer (Thermo Electron, Hemel Hempstead, UK) in ‘Top 10’ data-dependent acquisition mode. Raw files describing these mass spectra have been deposited at the Mass spectrometry Interactive Virtual Environment (MassIVE; Center for Computational Mass Spectrometry at University of California, San Diego) and can be accessed at https://massive.ucsd.edu/ProteoSAFe/datasets.jsp using the MassIVE ID MSV000078740. For the purposes of this project, data were re-analyzed using MaxQuant 1.5.8.3 analysis software (Tyanova et al., 2016) using standard settings and the following parameters: label-free quantitation and the iBAQ algorithm (Schwanhäusser et al., 2011) enabled; enzyme: trypsin/P; variable modifications: oxidation (M) and acetyl (Protein N-ter); and fixed modifications: carbamidomethyl (C); digestion mode: semi-specific free N terminus. Peptide spectra were matched to custom databases containing the IAV WSN proteome (including full-length translations of all six reading frames), an edited version of the *Bos taurus* proteome (UP000009136; retrieved from UniProt on 16/05/2017) in which all instances of the ubiquitin sequence had been deleted, and a single repeat of the ubiquitin protein sequence.

#### DEFEND sequencing of IBV infected cells

DEFEND-seq was performed as previously described (Rialdi et al., 2017). Briefly, RNA was extracted from A549 cells infected with influenza B virus (B/Wisconsin/01/2010) for 8 hours using Trizol (Invitrogen) and subjected to DNase treatment (QIAGEN). 5μg of DNase treated RNA was then incubated with 10U of Tobacco Acid Phosphotase (Epicentre; 37°C, 1.5h) to remove mRNA 5′caps. Sodium periodate was then added (to 500mM) into the reaction to block the 3′OH. The reaction was then allowed to proceed for 1.5h at 4°C, before being blocked by the addition of 1/10 volume 1M L-lysine, and incubating for an additional 10min at room temperature. RNA was purified with 1.8X AMPure XP beads (Beckman Coulter). Barcoded with RNA adapters were then ligated to the 5′ends of RNAs overnight at 16°C. Adaptor-ligated RNA was purified using 1.8X volume of AMPure XP beads. Ribosomal RNAs were removed using the Ribo-Zero Gold rRNA Removal Kit (Human/Mouse/Rat) (Illumina), according to the manufacturer’s protocol. cDNA synthesis was performed using a custom 3′ primer ((5′-AGA CGT GTG CTC TTC CGA TCT N^∗^N^∗^N^∗^N^∗^N^∗^N^∗^-3′, Bioo Scientific, N^∗^ = randomized bases) for 2 min at 65°C. Illumina adapters were added by PCR, and products were size-selected (200-400bp) using BluePippin 2% M1 gels (Sage Scientific). The library was validated on the Agilent Bioanalyzer, and samples were sequenced on the Illumina HiSeq 2500 platform in a 100bp SE read run format.

#### Preparation of CAGE libraries from LASV infected cells

Vero cells (ATCC-CCL81) grown on T75 flask were infected with recombinant LASV (Josiah strain) at MOI 0.1. At 2 days post infection, cells were lysed in Trizol (Invitrogen). The infection work with pathogenic Lassa virus and RNA lysate preparation were performed at the BSL4 facilities in Galveston National Laboratory in the University of Texas Medical Branch in accordance with institutional health and safety guidelines and federal regulations. Total RNA from the trizol-treated lysates was isolated and DNase treated using the Purelink RNA Minikit (Invitrogen). The purified RNA was then submitted for CAGE-sequencing at Kabushiki Kaisha DNAFORM, Japan.

#### Mouse Infection studies

All mice infection procedures were performed following protocols approved by the Icahn School of Medicine at Mount Sinai Institutional Animal Care and Use Committee (IACUC). Animal studies were carried out in strict accordance with the recommendations in the Guide for the Care and Use of Laboratory Animals of the National Research Council. Six to eight-week-old female BALB/c mice were obtained from Jackson Laboratories (Bar Harbor, ME). Mice were anesthetized by intraperitoneal injection of a mixture of 85mg/kg ketamine and 12.5mg/kg xylazine before infection before being inoculated intranasally with 50μl virus re-suspended in PBS. Mice were monitored daily for clinical signs of illness and weight loss after infection. Upon reaching 75% of initial body weight, animals were humanely euthanized with carbon dioxide (CO_2_) as per the IACUC protocol.

#### Preparation of RNA sequencing Libraries (Infected Mice)

3 mice were intranasally (i.n.) infected with 100 plaque-forming units (PFU) of viruses in a volume of 50 μL and euthanized at 6 days post-inoculation (d.p.i.). The middle lobe of the lung was collected for total RNA extraction, and the post-caval lobes of the lung was collected to determine virus titers by plaque assay on MDCK cells. Lung tissue was then homogenized in Trizol (Invitrogen), and RNA was extracted as per manufacturer’s guidelines. Libraries were constructed using the Illumina TruSeq Stranded Total RNA Library Prep Kit.

#### SIINFEKL expression analysis

For T cell activation assays with PB1-UFO(SIIN) and PB1-SIIN viruses, OT-I T cells were harvested from the spleen and lymph nodes of OTI transgenic mice and purified on the AutoMACS with the CD8a+ T Cell Isolation Kit (Miltenyi, Germany). DC2.4 cells were infected with influenza A viruses for 18 hours, and then co-cultured with OTI T cells. T cells were stained with anti-CD25 and anti-CD28 labeled antibodies at 24 hours post co-culture for activation assays. T cell proliferation assays were conducted at 48 hours post infection by measuring CellTrace Violet staining by flow cytometry.

For T cell activation assays with the NS-UFO(SIIN) and NA-SIIN viruses, IAV antigen was propagated by infecting MDCK cells with IAV PR8 wild-type, PR8 containing an NS segment with SIINFEKL inserted into frame 3 (PR8-NS.F3.SIIN) or PR8 containing an NA segment with SIINFEKL inserted into frame 1 (PR8-NA.SIIN) (Bottermann et al., 2018). The IAV antigen preparations were prepared as described (Stuller et al., 2010, Westerhof et al., 2019). Briefly, MDCK cells were infected for 48 h with each IAV stain and then centrifuged, resuspended in 0.1 M glycine buffer containing 0.9% NaCl (pH 9.75), and shaken at 4°C for 20 min. Preparations were sonicated 4 times at 10 s intervals before centrifugation, and the supernatant stored at −80°C.

Bone marrow was then taken from 10-14 week old naive female C57BL/6 mice, purchased from Envigo (UK) and maintained at the University of Glasgow under standard animal husbandry conditions in accordance with UK home office regulations and approved by the local ethics committee. Bone marrow derived dendritic cells (BMDCs) were prepared as previously described (Westerhof et al., 2019). Briefly, the tibias and femurs were flushed to obtain bone marrow cells. Red blood cells were lysed. Cells were then cultured in RPMI with 10% FCS, 100ug/ml penicillin-streptomycin and 2mM L-glutamine, in the presences of GM-CSF (prepared from X-63 supernatant), for 7 days, with media supplemented on day 2 and replaced on day 5. DCs were then harvested and incubated overnight with IAV antigen preparations. Control BMDCs were incubated with SIINFEKL peptide (Ovalbumin (257-264), chicken, Sigma-Aldrich) for 1 h at 37°C.

Lymph nodes (LN) (inguinal, brachial, axillary and cervical) and spleen were obtained from OTI mice sacrificed at weeks 12-13. CD8 T cells were negatively selected from LN and spleen using EasySep Mouse CD8+ T Cell Isolation Kit (Stemcell technologies).

BMDCs that had been exposed to viral antigen were co-cultured with CD8+ OTI T cells for 24 h. Activated T cells were detected by immunostaining with antibodies against Va2-E450 (Thermo Fisher), Vb5-PE (M59-4 BD Biosciences), CD8-Alexaflor488 (53-6.7 Thermo Fisher), CD25-APC (PC61.3 Thermo Fisher), CD44-PerCpC5.5 (IM7 Thermo Fisher), and CD69-PerCy7 (H1.2F3 Thermo Fisher). Data were acquired with a BD Fortessa cell analyzer and analyzed by FlowJo (BD, version 10).

#### Minireplicon Assays

Minireplicon assays were performed as previously described (Rezelj et al., 2019, Tilston-Lunel et al., 2017). Briefly, and using the plasmids indicated above, LT-1 transfection reagent (Mirus) was used to transfect sub-confluent BSR-T7/5 cells. After 24 h cells were processed using a Dual-Luciferase Reporter Assay System (Promega), with luciferase measured using Glowmax 20/20 luminometer (Promega).

### Quantification and Statistical Analyses

#### Mouse Infection Studies

Statistical significance between survival curves were compared using Log-rank (Mantel-Cox) test using Graphpad Prism 8.0 software. Two tailed Student’s t tests under the assumption of equal variances between groups were used to compare weight loss in mice from different groups for each day post infection. Data are shown as +/- SEM.

#### Quantitative qPCR assays

qPCR assays were done with 4 biological replicates (4 infected mice/condition). Statistical significance in gene expression was calculated with Graphpad Prism 8.0 software, and determined using one-tailed Student’s t test under the assumption of equal variances between groups. Data are shown as mean +/- SEM.

#### CAGE sequencing of WSN IAV virus infected cells

The sequencing of cap-snatched leader sequences was described in detail in a recent publication (Clohisey et al., 2020). Briefly, primary CD14+ human monocytes were isolated from 4 volunteer donors under ethical approval from Lothian Research Ethics Committee (11/AL/0168) and cultured in the presence of 100 ng/ml (104 U/ml) recombinant human colony-stimulating factor 1 (a gift from Chiron, USA) for 8 days to differentiate them into macrophages. Monocyte-derived macrophages were then infected with influenza (Udorn) at an MOI of 5, harvested at 0, 2, 7 and 24 hours post-infection (times defined as starting after a 1h adsorption step), and processed for RNA extraction using a miRNeasy Mini Kit (QIAGEN). Cap analysis of gene expression (CAGE) was performed as part of the FANTOM5 project, following the procedure of (Takahashi et al., 2012). Data were processed as in (Forrest et al., 2014) using custom Python scripts available at https://github.com/baillielab/influenza_cage 'ATG analysis.' The datasets analyzed during the current study are available in the Fantom5 repository, https://fantom.gsc.riken.jp/5/data/

#### Ribosome sequencing analyses

Footprints were obtained by first removing the AGATCGGAAGAGC linker and filtering for low quality sequences with Cutadapt (Martin, 2011). Contigs were then generated from the paired end reads with PANDASeq (Masella et al., 2012) using default parameters. Concurrent demultiplexing of the libraries by sample ID and UMI extraction was then performed. Reads were then aligned against rRNA and tRNA sequences with Bowtie (Langmead et al., 2009) to remove these contaminating sequences. Unmapped reads were aligned against a custom reference containing the human genome (hg38) and the eight genome segments of PR8 with HISAT2 (Kim et al., 2015). Host primer sequences were extracted from this alignment as well as unmapped reads by searching for a match to conserved nucleotides at the 5′ end of the influenza mRNA (GC[GA]AAAGCAGG). These reads were kept if the sequences could be extended to unambiguously assign it to a segment. Finally, 5′ end mapping was performed on these and all reads mapping to PR8.

#### RNA sequencing Analyses

After adaptor removal with cutadapt (Martin, 2011) and base-quality trimming to remove 3′read sequences if more than 20 bases with Q < 20 were present, paired-end reads were mapped to the mouse (mm10) reference genome with STAR (Dobin et al., 2013), and gene-count summaries were generated with featureCounts (Liao et al., 2014). DESeq2 (Love et al., 2014) was used to variance-normalize the data before a 1-factor model (gene ∼ConditionTimeMutant) was applied to identify differentially expressed genes. Differentially expressed genes were identified as genes that had a 2-fold difference, with an adjusted p .value < 0.01. RNA-seq raw data are deposited in GEO: GSE128519. Gene ontology analysis was performed using Metascape (Zhou et al., 2019).

#### LASV CAGE sequencing Analyses

Unique chimeric host-virus reads were extracted from the resulting FASTQ files by searching for a match to conserved nucleotides at the 5′ end of the LASV (Josiah Strain) mRNAs (GCAC[M]G[N]GGATCCT), allowing for a maximum of 1 mismatch, and removing all reads with ambiguously mapped nucleotides. The reference genome of LASV was obtained from UniProt (Accessions: J04324 and U73034). Reads were kept if at least 60 nucleotides could be mapped and assigned unambiguously to the viral reference sequences. Each read was then split into host derived or virus derived sequences based on the sequences of viral 5′ end (GCAC[M]G[N]GGATCCT). To calculate potential uvORF length, each read was extended bioinformatically, based off the mapped genome segment and coding sense, and translated from the first AUG found in the read.

#### Sequence Randomization Model for PB1-UFO length

Influenza A PB1 nucleotide sequences were obtained from the NCBI database (Zhang et al., 2017). Only unique sequences containing complete 5′UTR regions were included. Sequences containing ambiguous nucleotides were excluded. Multiple sequence alignment was then performed by using MUSCLE (Edgar, 2004).

We then constructed a codon usage table for each individual nucleotide sequence. To run the random sequence model, each nucleotide sequence was translated into two protein sequences in the two translation reading frames of interest: the canonical PB1 open reading frame (Pr-ORF) and PB1-UFO frame (Pr-UFO). Pr-UFO was considered as the observed protein sequence. Based on the frequencies of synonymous codons within a codon usage table, each Pr-ORF was reverse translated into multiple random nucleotide sequences in the open reading frame (Nt-ORFs) 1,000 times. 1,000 Nt-ORFs were then translated into proteins in the UFO frame (Pr-UFOs) which were considered as the expected protein sequences and their protein lengths were computed. We used the length of observed Pr-UFO and the lengths of expected Pr-UFOs to calculate the z-score for each nucleotide sequence. In total, 3140 unique IAV PB1 (H3N2 only: 499) sequences were included in the analysis. From the z-scores, P values were calculated for the Pr-UFOs occurrence biases. A threshold of p < 0.05 was used for the prediction of the likelihood of IAV PB1 sequences that were able to be translated. Similar analyses were also performed for other genome segments.

#### Frequency Propagator Ratio Analysis

##### Sequence dataset

Our study was based on a dataset of 26,742 human influenza A/H3N2 sequences available from the GISAID database (Shu and McCauley, 2017), which contains 6,244 unique PB1 strains. For downstream analyzes, we included only sequences that are had a complete 5′ and 3′UTR.

##### Prediction of RNA secondary structure

We used the most abundant unique, full length PB1 nucleotide sequence as an input to predict RNA secondary structure. RNA secondary structure was predicted using RNAfold from the ViennaRNA Webserver (version 2.4.13) (Gruber et al., 2008), using the default settings to calculate the minimum free energy (MFE) structure of the PB1 segment RNA. The output structure was saved in a dot-bracket format, and used to partition nucleotides into probable loop and stem regions for downstream analyses.

##### Strain tree reconstruction

Our analysis was based on an ensemble of strain trees obtained from the PB1 sequence dataset described above. Such trees describe the genealogy of influenza strains resulting from an evolutionary process under selection *(**Strelkowa and Lässig, 2012**).* Trees were constructed with maximum-likelihood phylogenies using FastTree *(**Price et al., 2010**).* We used a general time-reversible model. We further refined the tree topology with RAxML *(**Stamatakis, 2014**).* Given the output topology, we reconstructed maximum-likelihood sequences and timing of internal nodes with the TreeTime package *(**Sagulenko et al., 2018**).*

##### Frequency Propagator Ratio analysis

A detailed discussion of this method has previously been presented in Strelkowa and Lässig (2012) and Luksza and Lässig (2014).

Briefly, for a given polymorphism time-series, the frequency propagator G(x) can be used as a statistical measure of selection. G(x) is defined as the conditional probability that a mutation class of interest, with an initial frequency of $x_{i}$, reaches a frequency of $x>x_{i}$ at a later point in time. This is estimated in our dataset as

$$G\left(x\right)=\frac{n\left(x\right)}{n}$$

where n(x) is the number of mutations that reach frequency x

and n is the total number of mutations

Data availability might vary, depending on the year of sequence collection (fewer data points are available in the earlier years). As such, to attain a more robust measure of selection, we use the ratio of propagators between our mutation class of interest, G(x), against a neutral reference class of mutations, $G_{0}\left(x\right)$, to calculate

$$g\left(x\right)=\frac{G\left(x\right)}{G_{o}\left(x\right)}$$

where,

G(x) is the likelihood a mutation in a given class reaches frequency, x
$G_{o}\left(x\right)$ is the likelihood a mutation in the neutral reference class of mutations reaches the same frequency x.

The frequency propagator ratio takes into account both numbers and histories of the mutation class of interest. It is a robust measure of selection because it is (a) largely independent of data entry frequency, and (b) insensitive to clonal expansion of mutations.

At the limit x=1, the propagator ratio g(x) reduces to g, where

$$g=\frac{d/n}{d_{0}/n_{0}}$$

and,

d is the # of mutations in our class of interest that reach fixation
n is the total # of mutations in the same class of interest
$d_{0}$ is the # of mutations in a neutral reference mutation class that reaches fixation
$n_{0}\;$is the total # of mutations in the same neutral reference mutation class.

Selection on a mutation class of interest can be inferred from the value of g. g<1 suggests evolutionary constraints (negative selection) on the mutation class of interest relative to the reference class, where a fraction (1−g) of the mutations are under negative selection. g>1 suggests that fixation of the mutation class of interest undergoes positive selection, and that at least a fraction (g−1)/g of that mutation class is beneficial. g≈1 suggests weak or heterogenous selection acting on the mutation class of interest, relative to that of the neutral reference class.

To quantify selection occurring across the PB1-UFO frame, we calculated mutation frequencies in the set of codons derived from the following three regions (R1-R3)

R1: sequences that encode the N-terminal of PB1-UFO and the viral 5′UTR
R2: sequences that encode the C-terminal of PB1-UFO and overlap with the N-terminal of PB1
R3: Sequences that encode for the C-terminal region of the main PB1 ORF and do not overlap with PB1-UFO.

We chose to use synonymous mutations in the main PB1 ORF (reading frame) in R3 as our neutral reference class to calculate $G_{0}\left(x\right)$, as we reasoned that the majority of such mutations evolve near neutrality.

To quantify selection on the N-terminal of PB1-UFO in R1, we calculated the G(x) for two classes of mutations: Those that changed (non-synonymous in PB1-UFO) or did not change (synonymous in PB1-UFO) the amino acid sequence of PB1-UFO. We used synonymous mutations occurring the PB1 ORF in R3 as our neutral reference class ($G_{0}\left(x\right)$). We found that g<1 for both cases, suggesting that mutations occurring in this region of PB1-UFO were not likely to be fixed over time, and mostly undergo negative selection, relative to our reference class.

To quantify selection on the C-terminal of PB1-UFO in R2, we again calculated the G(x) for mutations that changed (non-synonymous in PB1-UFO) or did not change (synonymous in PB1-UFO) the amino acid sequence of PB1-UFO. We used synonymous mutations occurring the PB1 ORF in R3 as our neutral reference class $\left(G_{0}\left(x\right)\right)$. We found that g≈1 for both cases, suggesting that mutations occurring in this region of PB1-UFO underwent heterogenous selection, relative to that of the reference class.

Since R2 mutations in PB1-UFO appear to undergo heterogeneous selection, we asked if selection occurring on the main PB1 ORF was a contributing factor. To do so, we calculated the G(x) for mutations that changed (non-synonymous in PB1) or did not change (synonymous in PB1) the amino acid sequence of PB1 in R2. Synonymous mutations occurring the PB1 ORF in R3 as our neutral reference class $\left(G_{0}\left(x\right)\right)$. Here we found that g>1 for synonymous mutations and g<1 for non-synonymous mutations, suggesting that mutations that do NOT alter the amino acid sequence of PB1 are preferentially fixed over time. This suggests to us that part of the reason why PB1-UFO is undergoing heterogeneous selection in R2 is that there is a requirement to maintain the protein sequence of PB1. This is not surprising, given that PB1 is an integral part of the viral RNA dependent RNA polymerase complex.

Finally, to interrogate the effect of RNA structure, we classified nucleotides as pairing or non-pairing based on the MFE structure (discussed above) calculated by RNAFold. We masked nucleotides that were predicted to base pair (“stem-forming”) from downstream analyses as we reasoned that mutations in these nucleotides are likely to affect both RNA structure AND protein sequence, thus confounding later interpretations of the data. Regions that were not predicted to base pair (“loop nucleotides”) were then used for downstream calculations of frequency propagator ratios. Mutation frequencies were calculated in the same regions (R1, R2 and R3) and reading frames (PB1-UFO versus PB1) as described above. We found that similar effects to before were found, suggesting that RNA structure was not a major contributor to the maintenance of the PB1-UFO frame.

Note: The absolute number of polymorphism histories that reach a given frequency are finite (since the tree is constructed over a defined period of time). This can give rise to sampling fluctuations. These sampling uncertainties are reported as error bars in our figures.

#### Epitope predictions for PB1-UFO

Analyses were done using NetMHC3.4 and NetMHC4.0 (Andreatta and Nielsen, 2016). Binders were filtered using KD threshold of 500 nM. The collection of viral MHC-I epitopes was downloaded from IEDB database and preformatted for BLAST usage (makeblastdb -in iedb.fasta -parse_seqids -dbtype prot). Predicted epitopes from PB1-UFO were BLASTed against IEDB and the human proteome. For comparison with viral antigens we used the following commands: blastp -db iedb.fasta -query antigens.fasta -outfmt “6 qseqid sseqid pident ppos positive mismatch gapopen length qlen slen qstart qend sstart send qseq sseq evalue bitscore” -word_size 3 -gapopen 32767 -gapextend 32767 -evalue 1 -max_hsps_per_subject 1 -matrix BLOSUM62 -max_target_seqs 10000000 -out antigens.iedb.blast.out. For comparison with human proteome we used the command: blastp -db human.proteome.fasta -query antigens.fasta -outfmt “6 qseqid sseqid pident ppos positive mismatch gapopen length qlen slen qstart qend sstart send qseq sseq evalue bitscore” -word_size 3 -gapopen 32767 -gapextend 32767 -evalue 1 -max_hsps_per_subject 1 -matrix BLOSUM62 -max_target_seqs 10000000 -out antigens.human.proteome.blast.out. To find perfect matches between predicted epitopes and human proteome or viral antigens, we used the last command. First, we preformatted the human proteome (ensemble archive from December 2016): lastdb -p human.proteome human.proteome.fasta. Then we used following command to compare epitopes to this database: lastal -f MAF -r 2 -q 1 -m 100000000 -a 100000 -d 15 -l 4 -k 1 -j1 -P 10 human.proteome antigens.netMHC.score.fasta > antigens.human.last.out. Finally, obtained results were processed with bash and python and finally analyzed in PRISM 8. Similar processing was performed with viral antigens.

## Supplemental Information

Table S1. Summary of IAV CAGE Sequencing Data, Related to Figures 1D and S1B MI: Mock infection, Ud: A/Udorn/72(H3N2) infection

Table S2. Tables for Mass Spectrometry Data, Related to Figures 4A, 4B, and S4A–S4C A. Table of peptides sequences and intensities identified by mass spec in infected cell lysates (related to Figure S4A) B. Table of peptides sequences and intensities identified by mass spectrometry in virions (related to Figure S4B) C. Table of peptides sequences and intensities identified by mass spectrometry in Heaton et al. (2016) (related to Figure S4C)

Table S3. Table Showing the Mutations Used to Generate Control and Mutant Viruses, Related to Figures 4E, 4F and S4D–S4F Green letters in column 3 show the relative position canonical start codon (ATG) with respect to the sequence shown, and red letters indicate the mutations that were inserted.

Table S4. RNA-Seq of Lungs of Infected Mice, Related to Figures 4F, S4G, and S4H A. Table of differentially expressed genes found in the lungs of mice infected by PR8;PB1-UFO^Δ^ or PR8;PB1-UFO^SYN^ viruses at day 6 post infection (related to Figure S4G) B. Table of primers used to validate gene expression

Table S5. Gene Ontology of Differentially Expressed Genes Identified in Table S4, as Calculated by Metascape, Related to Figures 4F and S4I

Table S6. Table of Epitope Predictions for PB1-UFO, Related to Figures 5G and 5H

Table S7. Percentages of uAUG-Containing CS Sequences in IBV and LASV, Related to Figures 6A–6D and S7A–S7D
